# The negative intelligence-religiosity link may be differentiated according to cognitive test *g*-loadings and (Christian) religious denominations: primary study and meta-analytical evidence

**DOI:** 10.3389/fpsyg.2026.1633400

**Published:** 2026-03-12

**Authors:** Florian Dürlinger, Jakob Pietschnig

**Affiliations:** Department of Developmental and Educational Psychology, Faculty of Psychology, University of Vienna, Vienna, Austria

**Keywords:** cognitive abilities, denominations, *g*-loadings, intelligence, religiosity, religious behaviors, religious beliefs

## Abstract

Negative intelligence and religiosity associations have been well established. Despite a broad consensus about the direction of this effect, reported effect strengths differed substantially in previous research. Because measurement modalities regarding religiosity and intelligence have been found to explain some of the identified inconsistencies, the literature would benefit from thoroughly assessing both constructs. Moreover, little is known about the role of the influence of the *g*-loadedness of an intelligence test or the role of religious denominations. Most proposed explanations suggest that the intelligence and religiosity association is on *g*, rather than specific cognitive abilities. However, systematic empirical evidence on the generality of religiosity link with intelligence domains is currently unavailable and the potential causes of the intelligence and religiosity link are little understood. Here, we used subject-level data of American participants of the National Longitudinal Survey of Youth (NLSY) as well as a meta-analytical approach to examine test *g*-loading-specific effects on the intelligence and religiosity link. The primary data analyses (for two religiosity measures: *η*^2^ = 0.49 and *η*^2^ = 0.54) as well as the meta-analytical approach (*r* = 0.12 for poor vs. *r* = −0.20 for excellent *g*-loadedness) yielded indications of more pronounced intelligence and religiosity associations for studies assessing intelligence with more *g*-loaded tasks, thus tentatively suggesting a causal direction of intelligence influences on religiosity. Moreover, results of our primary data analyses suggest stronger associations of intelligence with religious beliefs than with religious behaviors in Catholic (*r* = −0.09 vs. *r* = 0.04), but not in Protestant (*r* = −0.06 vs. *r* < 0.01) participants.

## Introduction

Negative associations of intelligence and religiosity were initially reported about a century ago (Howells, 1928; Sinclair, 1928) and have been consistently replicated in numerous studies (Dürlinger and Pietschnig, 2022; Zuckerman et al., 2013, 2020). This link has been shown to generalize across various methodological approaches and sample characteristics in terms of effect direction, but not always in terms of effect strength (Dürlinger and Pietschnig, 2022). In empirical studies of this nature, religiosity is typically understood as the degree of involvement in some or all facets of religion and often operationalized in single item assessments, requiring individuals to provide information about praying behavior, participation in certain religious rituals, or their general self-reported belief (Zuckerman et al., 2013). We therefore refer to outcomes of such assessments henceforth as religiosity.

Accordingly, the scientific approach identifies external and internal behavioral patterns in religion that can be presently measured. Spirituality is assumed to represent a distinct construct in the scientific literature, thus indicating that certain aspects of religiosity are not assessed by the presently examined outcomes of interest. In other words, the empirical nature of this article (and others in this field) necessarily limits interpretations to operationalizable constructs.

While a number of moderating variables such as cognitive or religiosity measurement modality have been observed to affect the strength of the intelligence and religiosity association, meta-analyses have identified a substantial amount of unobserved heterogeneity that has not been explained by these variables and needs to be accounted for (Dürlinger and Pietschnig, 2022). The main goal of the present study is to assess test *g*-loadings and religious denomination as candidate causes for the between-studies strength differentiation of the intelligence and religiosity link.

### Effect-strength differentiation

So far, particularly cognitive ability and religiosity measurement modalities have been shown to yield a substantial differentiation in effect strength.

For instance, more pronounced associations were observed for directly assessed religious beliefs compared to religious behaviors (e.g., church attendance or participation in religious group activities; see Dürlinger and Pietschnig, 2022) and may be attributable to religious behaviors representing a rather noisy measure of personal beliefs. Other, more secular factors like social acceptance or social pressure (Allport and Ross, 1967) might play a role in exhibiting religious behaviors, rather than a profound belief in a certain religious dogma. Explicitly distinguishing between religious beliefs and behaviors is therefore necessary when it comes to assessing associations with intelligence. Differences in strength of intelligence and religious beliefs vs. intelligence and religious behaviors correlations, however, may conceivably be contingent on different religious denominations that exhibit different religious customs. For instance, religious rituals as well as an embeddedness of believers in the community are part of the religious identity for Catholicism and Judaism (Cohen et al., 2005). However, religious practices including other people might be less characteristic for American Protestantism. Due to the relatively low status of religious practices in Protestantism, their associations with intelligence may accordingly be less pronounced than in Catholicism, Judaism, or Islam.

In a similar vein, correlations with religiosity have been shown to be smaller in strength if intelligence was assessed via proxies like educational achievement (such as Grade Point Averages or Scholastic Aptitude Test scores) in contrast to psychometrically assessed IQ. This is most likely due to school or college grades being a less salient indicator of intelligence than IQ tests (Borghans et al., 2016). However, even in studies in which formal psychometric IQ tests were used, effect strengths of the IQ and religiosity link appeared to be often differentiated according to the assessed intelligence domains. For instance, religious beliefs appeared to yield stronger correlations with the performance on matrices tests (*r* = −0.26; Cavojová et al., 2019) than on vocabulary tests (*r* = −0.12; Kanazawa, 2010) or composite scores of IQ test batteries (Groninger Intelligence Test: *r* = −0.12; Verhage, 1964). However, potential influences of intelligence subdomain, test *g*-loadings, or number of subtests in IQ-test batteries have not yet been formally assessed.

Stronger associations of intelligence subtests with larger compared to those with smaller *g*-loadings with a given variable are difficult to detect in primary studies, even in cases where test batteries with several subtests are administered because such systematic effects cannot readily be disentangled from (sub)test-specific effects within a given test battery. However, meta-analytical approaches allow for a formal identification of such effects (e.g., Pietschnig et al., 2022) although to date no such evidence is available for the intelligence and religiosity link. To identify the meaning, causes, and nature of the intelligence and religiosity link, it is necessary to assess whether this association relates to general intelligence or (a subset of) specific abilities.

### Potential causal mechanisms

To date, several potential causal mechanisms have been suggested to explain the causes for the negative intelligence and religiosity link. For instance, analytic thinking styles have been shown to be positively associated with intelligence (Frederick, 2005) but negatively with religiosity (Gervais and Norenzayan, 2012) and thus have been proposed to act as a mediator between these two variables. Furthermore, religiosity has been suggested to satisfy certain psychological needs and desires as indicated by positive associations with self-regulation and self-control (McCullough and Willoughby, 2009) or beliefs in compensatory control (Sedikides and Gebauer, 2010). Intelligence on the other hand has also been linked to self-control (Shamosh and Gray, 2008) and control beliefs (Miller and Lachman, 2000). Common attributes like these support the idea that intelligence and religiosity may be functionally equivalent to a certain extent (Zuckerman et al., 2013), thus decreasing the necessity for religiosity in more intelligent individuals. In a different vein, it has been hypothesized that less intelligent individuals may be more susceptible to adopting belief systems upheld by their religious surroundings because intelligence is negatively associated with conformity (Rhodes and Wood, 1992).

**Figure 1:**
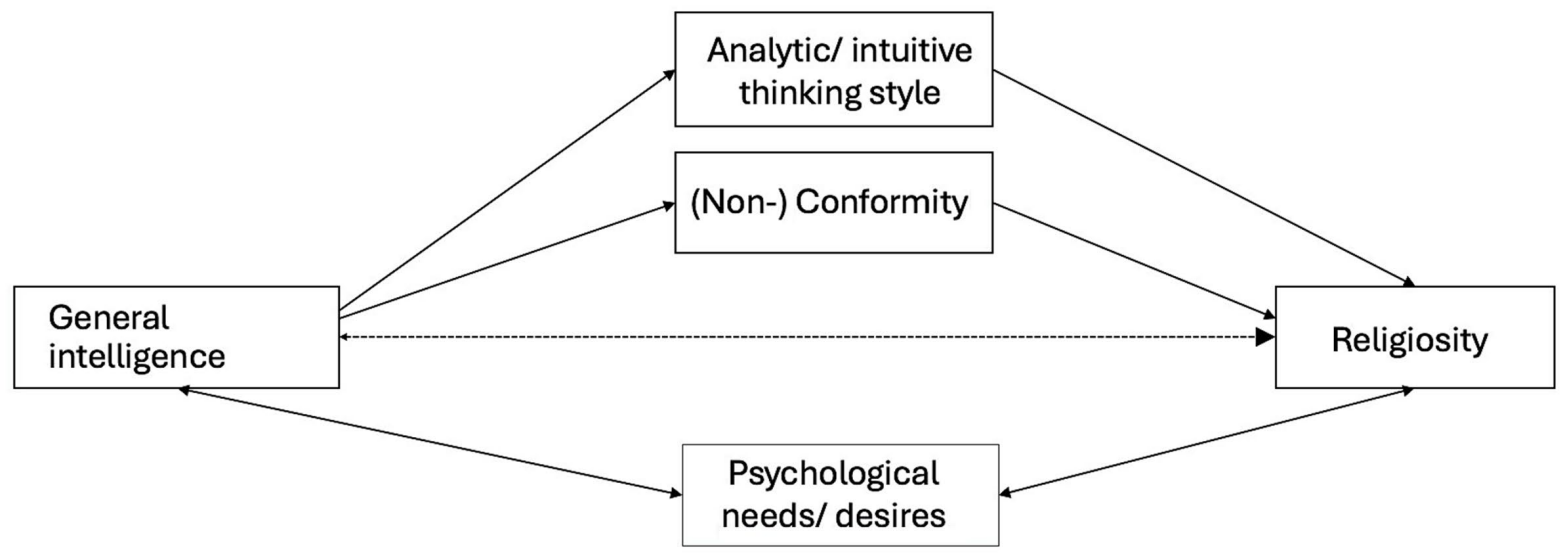
Proposed causal mechanisms of intelligence and religiosity associations. Depicted is a conceptual diagram, not a statistical model. While thinking styles and conformity have been proposed to mediate effects of intelligence on religiosity, psychological needs and desires may be fulfilled to a certain extent by intelligence and religiosity and therefore may exhibit interactions with both constructs.

Figure 1 illustrates a conceptual diagram of the proposed pathways linking cognitive styles, psychological needs, conformity and religiosity.

All of these proposed candidate theories are consistent with the assumption that religiosity should be more substantially associated with stronger rather than lower *g*-loaded tests. However, results from recent empirical studies do not unequivocally support these ideas with some studies yielding the expected positive (Dutton and Kirkegaard, 2021) but others negative relations of the intelligence and religiosity link with *g* (Dutton et al., 2020).

Thus, in the light of the available ambiguous evidence, the role of test *g-*loadedness for the intelligence and religiosity association as well as the generality of this link across different religious denominations remains unclear.

### The present study

Here, we examine whether intelligence and religiosity associations are differentiated according to intelligence test *g*-loadings. To this end, we use both primary data analyses as well as a meta-analytical approach. First, we use archival data of American participants of two cohorts of the National Longitudinal Survey of Youth (NLSY) to assess cross-sectional associations of intelligence and academic achievement with religiosity. On the other hand, we use a meta-analytical approach to examine test *g*-loading-specific effects on the intelligence and religiosity link based on all studies from the available literature. In addition, we investigate potential differences in strength of intelligence and religious beliefs vs. intelligence and religious behaviors associations in different religious denominations. The entire study protocol including all hypotheses and planned confirmatory analyses were preregistered prior to data analyses on the Open Science Framework at https://osf.io/abzvy (see Supplementary File S1 for deviations from the preregistered protocol).

### Research questions and hypotheses

First, we expect religious beliefs and religious behaviors to be positively correlated in our primary data analyses. Second, for the primary data as well as the meta-analytical approach intelligence is expected to be negatively correlated with religious beliefs as well as with religious behaviors, respectively. However, we hypothesize that associations of intelligence and religious behaviors will be less pronounced than those of intelligence and religious beliefs (in subject level and meta-analytical data). Third, tasks with higher *g*-loadings are expected to be correlated more strongly with religious beliefs than tasks with lower *g*-loadings. Similarly, higher *g*-loaded tasks are expected to be correlated more strongly with religious behaviors than less *g*-loaded tasks (in subject level and meta-analytical data). Finally, in our primary data analyses we expect differences between effect size strength of correlations of intelligence and religious beliefs and intelligence and religious behaviors to be larger in Protestant participants than in Catholic, Jewish or Muslim participants.

## Study 1

### Materials and methods

#### Sample

For our primary data analyses, our hypotheses testing was based on US-American subject-level data from two cohorts of the National Longitudinal Survey of Youth (NLSY; https://www.nlsinfo.org, accessed on 7 July 2024), one first interviewed in 1979 (NLSY79) and one in 1997 (NLSY97). The NLSY is a nationally representative longitudinal survey of noninstitutionalized young men and women born during the years 1957 through 1964 (NLSY79) or born between 1980 and 1984 (NLSY97) and living in the United States when the survey began. It has been conducted annually or biannually since 1979 or 1997, respectively. In the NLSY79 cohort participants’ (*N* = 12,686) age averaged 17.9 years (SD = 2.3) and recruited samples were balanced in terms of sex (6,283 women; 49.5%). In the NLSY97 cohort participants’ (*N* = 8,984) age averaged 14.31 years (SD = 1.5) and recruited samples were balanced in terms of sex (4,385 women; 48.8%).

### Measures

#### Cognitive abilities

In the NLSY79 cohort, the Armed Services Vocational Aptitude Battery (ASVAB) was administered. The ASVAB is a speeded multi-aptitude test measuring the respondent’s knowledge and skills in 10 subscales: Arithmetic Reasoning (assessing the ability to solve verbalized arithmetic problems), Numerical Operations (assessing the ability to solve simple mathematical calculations), General Science (assessing knowledge of physical and biological sciences), Paragraph Comprehension (assessing the ability to obtain information from written passages), Mathematics Knowledge (assessing the knowledge of high school mathematics principles), Coding Speed (assessing the ability to find a number quickly and accurately from a table of numbers), Mechanical Comprehension (assessing the knowledge of mechanical and physical principles), Word Knowledge (assessing the ability to select the correct meaning of words presented in context and to identify the best synonym for a given word), Electronics Information (assessing the knowledge of electricity and electronics), Auto and Shop Information (assessing the knowledge of automobile technology as well as of tools and shop terminology and practices). In the NLSY97 cohort, a computerized adaptive version of the ASVAB (CAT-ASVAB) was administered. Subscale names and content of both ASVAB and the CAT-ASVAB are largely identical, but in the CAT-ASVAB Automotive and Shop Information is assessed by means of two separate subscales and Assembling Objects (assessing the ability to determine how an object will look when its parts are put together) has been added as another subscale. The NLSY provides ability estimates based on Item Response Theory (IRT) for all 10 or 12 subtests, respectively. A lower value indicates poorer and a larger one better performance.

Moreover, we obtained math and verbal scores from the Scholastic Aptitude Test (SAT), the American College Test (ACT) and the Preliminary Scholastic Aptitude Test (PSAT) from both cohorts. Finally for the NLSY79, IQ-scores of the following tests were included in our analyses: California Test of Mental Maturity (Sullivan et al., 1946), Otis-Lennon Test of Mental Maturity (Grotelueschen, 1969), Lorge-Thorndike Intelligence Test (Lorge and Thorndike, 1962), Henmon-Nelson Test of Mental Maturity (Henmon and Nelson, 1954), Kuhlmann-Anderson Intelligence Test (Kuhlmann and Anderson, 1927), Differential Aptitude Test (Bennett et al., 1947), Coop School & College Ability Test (Tempero and Ivanoff, 1960), Stanford-Binet Intelligence Scale (Roid and Pomplun, 2012), Wechsler Intelligence Scale for Children (Wechsler, 1949).

#### Religiosity

In the NLSY79 cohort, religiosity was assessed via two items: “In the past year, about how often have you attended religious services?” (More than once a week/about once a week/two or three times a month/about once a month/several times or less during the year/not at all?) and “What is your present religion, if any?” (Protestant/Roman Catholic/Jewish/None/Other). In the NLSY97 cohort, religiosity was assessed via several questions: “I don’t need religion to have good values” (True/False), “Religious teachings should be obeyed exactly as written in every situation” (True/False), “I often ask God to help me make decisions” (True/False), “God has nothing to do with what happens to me personally” (True/False), “I pray more than once a day” (True/False) and “What is your present religion, if any?” (Protestant/Roman Catholic/Jewish/None/Other). Because religious attendance was not assessed in the NLSY97 cohort, we treat praying as a measure of religious behaviors here whilst we consider the other four indices as measures of religious beliefs.

### Analyses

#### Religious beliefs and behaviors

We obtained Spearman-rank correlation coefficients for all ordinally scaled variables. To facilitate interpretation and in order to conduct formal comparisons, all Spearman correlation coefficients were transformed into Pearson coefficients via Pearson’s conversion formula (Pearson, 1907): *r*_p_ = 2 *sin(*r*_s_ * (*π*/6)). To assess potential differences in strength of intelligence and religious beliefs vs. intelligence and religious behaviors correlations, Fisher’s *z* transformed values of the respective associations were obtained and subsequently formally compared (Myers and Sirois, 2006). For the NLSY97-cohort, we averaged Fishers *z*-transformed associations of all four indices of religious beliefs and correlated these scores with the respective CAT-ASVAB subtests. The resulting 12 correlations were averaged to obtain a single coefficient for this intelligence and beliefs association. Overall correlation strengths of intelligence and religious beliefs were then formally compared with those of intelligence and religious behaviors.

Pearson correlations for the association of religious beliefs or behaviors and scores on the achievement measures (ACT, SAT, PSAT) were obtained for the NLSY79 cohort. Formal tests for differences in strength were conducted with corresponding Fisher’s *z* values.

For each denomination (Protestants, Catholics, Jews, Muslims) Pearson correlations for the association of intelligence and religious behaviors as well as NLSY97-based religious beliefs (i.e., after averaging the four religious beliefs indices) were obtained. For formal comparisons of correlation strengths, Fisher’s *z*-transformed values were used.

#### *g*-loadings

To obtain *g*-loadings from the ASVAB and CAT-ASVAB subtests, we subjected the ability estimates of the 10 (ASVAB) or 12 (CAT-ASVAB) subtests to an unrotated principal component analysis (see Revelle and Wilt, 2013). The subtest’s loading on *g* is represented by the subtests’ loadings on the first unrotated factor. Then, we calculated Pearson correlations of scores of the respective subtests and religious beliefs or behaviors. The associations of the subtest scores and the religiosity indices were regressed on the *g*-loadings of the respective subscale.

The main analyses were conducted with the original ordinal scaling of the item asking about frequency of attendance to religious services. We provide supplemental group comparisons by dichotomizing it into attendance (More than once a week/about once a week/two or three times a month/about once a Month/several times or less during the year) and no attendance (not at all), following the approach by Dürlinger et al. (2023) (Supplementary File S2 at https://osf.io/qgfcy/files/h9q8a).

We interpret the Pearson correlation coefficients according to Funder and Ozer’s effect size classification (Funder and Ozer, 2019), where absolute *r* = 0.05, 0.10, 0.20, and 0.30 values are considered to represent the bottom thresholds of very small, small, moderate, and large effects, respectively. All analyses were performed in the open-source software R4.3.2 using the packages “factoextra” (Kassambara and Mundt, 2020) and “metafor” (Viechtbauer, 2010). Our entire analytic code is available at https://osf.io/w2u5e.

#### Exploratory analyses

For the NLSY79 cohort, measures of religiosity were correlated with IQ-scores of nine intelligence test batteries (i.e., California Test of Mental Maturity, Otis-Lennon Test of Mental Maturity, Lorge-Thorndike Intelligence Test, Henmon-Nelson Test of Mental Maturity, Kuhlmann-Anderson Intelligence Test, Differential Aptitude Test, Coop School & College Ability Test, Stanford-Binet Intelligence Scale, Wechsler Intelligence Scale for Children).

### Results

#### NLSY79

##### Religious beliefs and behaviors

For the NLSY79 cohort, Pearson correlations of religious attendance and all ASVAB-subtests are shown in Table 1, correlations of academic achievement in Table 2, and correlations of other intelligence measures in Table 3. Religious attendance was negatively correlated with General Science (*r* = −0.05), Word Knowledge (−0.04), Auto and Shop Information (*r* = −0.15), Mechanical Comprehension (*r* = −0.07), and Electronics Information (*r* = −0.11). Associations with Numerical Operations, Coding Speed, and Mathematics Knowledge were positive and significant but trivial in strength (*r*-values = 0.023 to 0.044). Moreover, religious attendance was unrelated to Arithmetic reasoning (*r* = −0.014, *p* > 0.05) and Paragraph Comprehension (*r* = −0.006, *p* > 0.05). All academic achievement measures were significantly negatively associated with religious attendance (*r*-values = −0.061 to −0.128; all *p*-values < 0.05), excepting ACT-scores which were uncorrelated with religiosity (*r* = −0.023 for math scores and *r* = 0.009 for verbal scores, *p*-values > 0.05). In our exploratory analyses (Table 3), only the Wechsler Intelligence Scale (*r* = −0.38) and the Differential Aptitude Test (*r* = 0.12) showed meaningful associations with religious attendance. In all, religious attendance showed small, mostly negative relations to intelligence measures, conforming to our expectations.

**Table 1:** Correlations of ASVAB subtests and religious attendance for the 1979 cohort.

	General science	Arithmetic reasoning	Word knowledge	Paragraph comprehension	Numerical operations	Coding speed	Auto and Shop information	Mathematics knowledge	Mechanical comprehension	Electronics info	Religious attendance	Age
General Science	—											
Arithmetic Reasoning	0.741^***^	—										
Word Knowledge	0.818^***^	0.737^***^	—									
Paragraph Comprehension	0.711^***^	0.697^***^	0.797^***^	—								
Numerical Operations	0.550^***^	0.636^***^	0.624^***^	0.617^***^	—							
Coding Speed	0.498^***^	0.549^***^	0.589^***^	0.588^***^	0.720^***^	—						
Auto and Shop Information	0.653^***^	0.552^***^	0.569^***^	0.480^***^	0.358^***^	0.313^***^	—					
Mathematics Knowledge	0.716^***^	0.819^***^	0.714^***^	0.687^***^	0.644^***^	0.553^***^	0.450^***^	—				
Mechanical Comprehension	0.726^***^	0.698^***^	0.650^***^	0.585^***^	0.451^***^	0.408^***^	0.734^***^	0.623^***^	—			
Electronics Info	0.781^***^	0.678^***^	0.721^***^	0.628^***^	0.468^***^	0.417^***^	0.744^***^	0.614	0.752^***^	—		
Religious Attendance	−0.052^***^	−0.014	−0.042^***^	−0.006	0.023^*^	0.024^*^	−0.147^***^	0.044^***^	−0.071^***^	−0.110^***^	—	
Age	0.193^***^	0.167^***^	0.255^***^	0.186^***^	0.133^***^	0.185^***^	0.232^***^	0.094^***^	0.170^***^	0.258^***^	−0.188^***^	—
Sex	−0.148^***^	−0.104^***^	0.022^*^	0.087^***^	0.112^***^	0.207^***^	−0.447^***^	−0.025^**^	−0.308^***^	−0.302^***^	0.109^***^	0.008

Cell entries are Pearson correlation coefficients for the total sample of the NLSY79 cohort. Correlations with the ordinal response format were originally calculated as Spearman coefficients and then transformed into Pearson coefficients. Sex: 0 = men; 1 = women. Religious attendance = Frequency of religious attendance. ^*^p < 0.05 and ^***^p < 0.001.

**Table 2:** Correlations of academic achievement scores and religious attendance for the 1979 cohort.

	SAT math	SAT verbal	ACT math	ACT verbal	PSAT math	PSAT verbal	Religious attendance	Age
SATmath	—							
SATverbal	0.748^***^	—						
ACTmath	0.860^***^	0.646^***^	—					
ACTverbal	0.723^***^	0.738^***^	0.676^***^	—				
PSATmath	0.863^***^	0.701^***^	0.823^***^	0.648^***^	—			
PSATverbal	0.660^***^	0.860^***^	0.616^***^	0.724^***^	0.713^***^	—		
Religious attendance	−0.109^**^	−0.128^***^	−0.023	0.009	−0.061^*^	−0.080^**^	—	
Age	−0.022	0.030	0.051	0.037	0.004	0.048	−0.188^***^	—
Sex	−0.260^***^	−0.114^***^	−0.222^***^	0.025	−0.196^***^	−0.082^**^	0.114^***^	0.008

Cell entries are Pearson correlation coefficients for the total sample of the NLSY79 cohort. Correlations with the ordinal response format were originally calculated as Spearman coefficients and then transformed into Pearson coefficients. Sex: 0 = men; 1 = women. SAT, Scholastic Aptitude Test; ACT, American College Test; PSAT, Preliminary Scholastic Aptitude Test. Religious attendance = Frequency of religious attendance. ^*^p < 0.05, ^**^p < 0.01, and ^***^p < 0.001.

**Table 3:** Correlations of intelligence measures with religious attendance for the 1979 cohort.

	California	Otis	Lorge	Henmon	Kuhlmann	DAT	Coop	Stanford	Wechsler	Religious attendance	Age
California	—										
Otis	0.309^**^	—									
Lorge	0.592^***^	0.605^***^	—								
Henmon	0.205	0.846^***^	0.354	—							
Kuhlmann	0.169	0.729^***^	0.849^***^	0.270	—						
DAT	0.666^***^	0.369^***^	0.340^***^	0.268	0.448	—					
Coop	−0.096	0.706^***^	0.273	0.666^**^	0.224	0.393^*^	—				
Stanford	0.028	0.389	−0.101	0.534^*^	0.157	0.861	—	—			
Wechsler	0.698^*^	0.780^***^	0.075	—	0.098	0.839^*^	—	0.806^***^	—		
Religious attendance	−0.018	0.010	−0.014	0.004	0.142	0.119^**^	0.059	−0.007	−0.376^***^	—	
Age	0.056	0.062^*^	0.022	0.172^*^	0.153^*^	−0.047	0.191^*^	−0.117	0.037	−0.188^***^	—
Sex	0.005	0.054	0.133^***^	−0.005	−0.035	−0.020	−0.010	−0.095	−0.019	0.114^***^	0.008

Cell entries are Pearson correlation coefficients for the total sample of the NLSY79 cohort. Sex: 0 = men; 1 = women. Religious attendance = Frequency of religious attendance. California: California Test of Mental Maturity, Otis: Otis-Lennon Test of Mental Maturity, Lorge: Lorge-Thorndike Intelligence Test, Henmon: Henmon-Nelson Test of Mental Maturity, Kuhlmann: Kuhlmann-Anderson Intelligence Test, DAT, Differential Aptitude Test, Coop, Coop School & College Ability Test, Stanford, Stanford-Binet Intelligence Scale; Wechsler, Wechsler Intelligence Scale for Children. ^*^p < 0.05, ^**^p < 0.01, ^***^p < 0.001.

##### Denomination

We obtained average correlations of the 10 ASVAB-subtests and religious attendance for each denomination separately. Associations of intelligence and religious attendance were stronger (*z* = 1.936, *p* = 0.026) for Protestants than for Catholics (*r* = −0.05 vs. -0.01). Intelligence and religious attendance associations for Jewish participants (*r* = 0.07) did not differ from those of Catholics (*z* = −0.807, *p* = 0.21) or Protestants (*z* = 1.212, *p* = 0.113). However, this might be due to the comparatively low number of Jewish participants in this cohort (*n* = 103). Muslim participants had to be omitted due to low case numbers. Numerical details for denomination-specific associations of intelligence and religiosity are provided in Supplementary File S3 at https://osf.io/rzhuj.

##### *g*-loadings

Regressing associations of the subtest scores and religious involvement on *g*-loadings yielded no significant associations (*β* = −0.693, *p* = 0.633, *R*^2^ = −0.091, *η*^2^ = 0.03; Figure 1), in contrast to our expectations. ASVAB-subtests *g*-loadings (in descending order) as well as their associations with respective religiosity measures are detailed in Table 4.

**Table 4:** *g*-loadings of ASVAB and CAT_ASVAB subtests.

Subtest (ASVAB) 1979	*g*-loading	*r* frequency of rel. attendance	Subtest (CAT_ASVAB) 1997	*g*-loading	*r* value	*r* obey	*r* decision	*r* with happens	*r* praying
General science	0.334	−0.052^***^	Mathematics Knowledge	0.351	−0.041	−0.143	−0.050	0.050	0.001
Word knowledge	0.333	−0.042^***^	General Science	0.346	−0.023	−0.124	−0.031	0.060	0.014
Paragraph comprehension	0.326	−0.006	Arithmetic Reasoning	0.346	−0.013	−0.132	−0.006	0.059	0.032
Arithmetic reasoning	0.325	−0.014	Paragraph Comprehension	0.339	0.031	−0.032	−0.023	0.055	0.036
Electronics info	0.322	−0.110^***^	Word Knowledge	0.321	−0.014	−0.034	−0.005	0.027	0.052
Mechanical comprehension	0.316	−0.071^***^	Assembling Objects	0.296	0.014	−0.040	0.003	0.038	−0.025
Mathematics knowledge	0.313	0.044^***^	Mechanical Comprehension	0.295	−0.013	−0.083	−0.013	0.022	0.038
Numerical operations	0.303	0.023^*^	Coding Speed	0.273	0.006	−0.171	−0.058	0.082	−0.055
Auto and shop information	0.301	−0.147^***^	Numerical Operations	0.266	<0.001	−0.188	−0.058	0.092	−0.048
Coding speed	0.286	0.024^*^	Electronics Info	0.250	−0.056	−0.077	−0.075	0.027	0.002
			Shop Information	0.165	−0.059	−0.117	−0.039	0.008	−0.095
			Auto Information	0.108	−0.097	−0.133	0.042	−0.091	0.012

The ASVAB-subtests are ordered descending in regards to the respective loading on the first factor of the principal component analysis. For the CAT-ASVAB, factor loadings were obtained via positive ability estimates. Value = “I do not need religion to have good values” (0 = True; 1 = False), Obey = “Religious teachings should be obeyed exactly as written in every situation” (0 = False; 1 = True), Decision = “I often ask God to help me make decisions” (0 = False; 1 = True), Happens = “God has nothing to do with what happens to me personally” (0 = True; 1 = False), Praying = “I pray more than once a day” (0 = False; 1 = True).

#### NLSY97

##### Religious beliefs and behaviors

Cross-sectional associations of the 12 CAT-ASVAB subtests and several indices of religiosity are provided in Table 5. In the NLSY97 cohort, religiosity was assessed via several items measuring religious beliefs and one item measuring religious behaviors (praying). In this cohort, praying was positively correlated with responses to the items “I do not need religion to have good values” (*r* = 0.199, *p* < 0.001); “I often ask God to help me make decisions” (*r* = 0.383, *p* < 0.001); “God has nothing to do with what happens to me personally” (*r* = 0.111, *p* < 0.001) and virtually unrelated with “Religious teachings should be obeyed exactly as written in every situation” (*r* = −0.034, *p* < 0.01). In all, this confirmed our expectations regarding positive associations of religious beliefs and religious behaviors.

**Table 5:** Correlations of CAT-ASVAB subtests and religiosity indices for the 1997 cohort.

	Science	Arithmetic	Vocabulary	Comprehension	Numeric	Speed	Auto	Shop	Mathematics	Mechanical	Electronics	Assembling	Age	Sex	Value	Obedience	Decision	Happening
Science	—																	
Arithmetic	0.453^***^	—																
Vocabulary	0.489^***^	0.347^***^	—															
Comprehension	0.362^***^	0.399^***^	0.403^***^	—														
Numeric	0.207^***^	0.356^***^	0.199^***^	0.238^***^	—													
Speed	0.201^***^	0.309^***^	0.233^***^	0.278^***^	0.615^***^	—												
Auto	−0.007	0.048	−0.073	−0.069	−0.143^*^	−0.150^*^	—											
Shop	0.093	0.085	0.035	0.136^**^	−0.003	0.024	0.371^***^	—										
Mathematics	0.425^***^	0.591^***^	0.359^***^	0.400^***^	0.450^***^	0.405^***^	−0.109	0.033	—									
Mechanical	0.404^***^	0.306	0.225^***^	0.154^***^	0.107^***^	0.124^***^	0.175^*^	0.287^***^	0.265^***^	—								
Electronics	0.417^***^	0.196^***^	0.242^***^	0.101^**^	0.046	0.070^*^	0.144	0.284^***^	0.179^***^	0.441^***^	—							
Assembling	0.235^***^	0.332^***^	0.201^***^	0.263^***^	0.238^***^	0.234^***^	−0.136	0.088	0.315^***^	0.199^***^	0.110^**^	—						
Age	0.202^***^	0.148^***^	0.174^***^	0.116^***^	0.265^***^	0.278^***^	0.088	0.122^**^	0.266^***^	0.188^***^	0.122^***^	0.113^***^	—					
Sex	−0.146^***^	−0.089^***^	−0.019	0.005	0.092^***^	0.161^***^	−0.152^*^	−0.146^***^	0.022	−0.199^***^	−0.152^***^	−0.090^***^	0.009	—				
Value	−0.023	−0.013	−0.014	0.031	<0.001	0.006	−0.097	−0.059	−0.041	−0.013	−0.056	0.014	0.010	<0.001	—			
Obedience	−0.124^**^	−0.132^**^	−0.034	−0.032	−0.188^***^	−0.171	−0.133	−0.117	−0.143^***^	−0.083	−0.077	−0.040	0.020	0.004	0.017	—		
Decision	−0.031	−0.006	−0.005	−0.023	−0.058^***^	−0.058^***^	0.042	−0.039	−0.050^*^	−0.013	−0.075	0.003	0.023	−0.003	0.273^***^	0.035^**^	—	
Happening	0.060^*^	0.059^*^	0.027	0.055^*^	0.092^***^	0.082^***^	−0.091	0.008	0.050^*^	0.022	0.027	0.038	0.018	0.016	0.237^***^	−0.084^***^	0.158^***^	—
Praying	0.014	0.032	0.052	0.036	−0.048^***^	−0.055^***^	0.012	−0.095	<0.001	0.038	0.002	−0.025	0.015	0.011	0.199^***^	−0.034^**^	0.383^***^	0.111^***^

Cell entries are Pearson correlation coefficients for the total sample of the NLSY97 cohort. Correlations with the ordinal response format were originally calculated as Spearman coefficients and then transformed into Pearson coefficients. Science = General Science, Arithmetic = Arithmetic Reasoning, Vocabulary = Word Knowledge, Comprehension = Paragraph Comprehension, Numeric = Numerical Operations, Speed = Coding Speed, Auto = Auto Information, Shop = Shop Information, Mathematics = Mathematics Knowledge, Mechanical = Mechanical Comprehension, Electronics = Electronics Info, Assembling = Assembling Objects. Sex: 0 = men; 1 = women. Value = “I do not need religion to have good values” (0 = True; 1 = False), Obedience = “Religious teachings should be obeyed exactly as written in every situation” (0 = False; 1 = True), Decision = “I often ask God to help me make decisions” (0 = False; 1 = True), Happening = “God has nothing to do with what happens to me personally” (0 = True; 1 = False), Praying = “I pray more than once a day” (0 = False; 1 = True). ^*^p < 0.05, ^**^p < 0.01, and ^***^p < 0.001.

Associations with intelligence were less straightforward for this cohort. For instance, intelligence was virtually unrelated to item-responses to “I do not need religion to have good values,” with the strongest correlations for Auto (*r* = −0.10) and Shop Information (*r* = −0.06). Numerical Operations (*r* = 0.092, *p* < 0.001) and Coding Speed (*r* = 0.082, *p* < 0.001) were positively associated with responses to the item “God has nothing to do with what happens to me personally.” Intelligence showed the strongest (and consistently negative) associations with responses to the item “Religious teachings should be obeyed exactly as written in every situation” (*r*-values = −0.032 to −0.188) and with responses to the item “I often ask God to help me make decisions” (*r*-values = 0.003 to −0.075). Correlating religious beliefs scores with the 12 CAT-ASVAB subtests yielded more consistent results (see Table 6). Associations of intelligence and religious beliefs were continuously negative, while intelligence was virtually unrelated with religious behaviors. As hypothesized, the overall correlation of intelligence and religious beliefs (*r* = −0.062, *p* = 0.002) was more pronounced (*z* = −1.908, *p* = 0.028) than the correlation of intelligence and religious behaviors (*r* = −0.003, *p* = 0.933).

**Table 6:** Formal tests for differences in strength of religious beliefs and intelligence associations with praying and intelligence associations with all CAT-ASVAB-subtests.

	Correlation with rel. beliefs (*N*)	Correlation with praying (*N*)	*z*
General Science	−0.073^***^ (2285)	0.014 (1488)	−2.608^**^
Arithmetic Reasoning	−0.017 (2382)	0.032 (1545)	−1.484
Word Knowledge	−0.056^*^ (1992)	0.052 (1265)	−2.983^**^
Paragraph Comprehension	−0.029 (2749)	0.036 (1745)	−2.118^*^
Numerical Operations	−0.111^***^ (6965)	−0.048^***^ (4791)	−3.405^***^
Coding Speed	−0.121^***^ (6755)	−0.055^***^ (4635)	−3.516^***^
Auto Information	−0.014^*^ (286)	0.012 (135)	−0.243
Shop Information	−0.096 (529)	−0.095 (336)	−0.012
Mathematics Knowledge	−0.106^***^ (3209)	0.001 (1995)	−3.748^***^
Mechanical Comprehension	−0.062^*^ (1552)	0.038 (1003)	−2.465^**^
Electronics Info	−0.023 (844)	0.002 (511)	−0.449
Assembling Objects	−0.034 (2341)	−0.025 (1516)	−0.259

Cell entries are Pearson correlation coefficients for the NLSY97 cohort. Correlations of the respective CAT-ASVAB subtests and four indices of religious beliefs: “I do not need religion to have good values” (0 = True; 1 = False); “Religious teachings should be obeyed exactly as written in every situation” (0 = False; 1 = True); “I often ask God to help me make decisions” (0 = False; 1 = True); “God has nothing to do with what happens to me personally” (0 = True; 1 = False) were averaged before formal comparisons in terms of strength with correlations of the CAT-ASVAB subtests and praying: “I pray more than once a day” (0 = False; 1 = True). ^*^p < 0.05, ^**^p < 0.01, and ^***^p < 0.001.

##### Denomination

Associations of intelligence and religious beliefs (*r* = −0.090, *p* = 0.019) were more pronounced than associations of intelligence and religious behaviors (*r* = 0.040, *p* = 0.366) in Catholic participants (*z* = −2.234, *p* = 0.013). In contrast, associations of intelligence and religious beliefs (*r* = −0.056, *p* = 0.046) did not differ from associations of intelligence and religious behaviors (*r* = 0.001, *p* = 0.976) in Protestant participants (*z* = −1.303, *p* = 0.096). However, intelligence and religious beliefs as well as religious behaviors did not significantly differ between Catholic and Protestant participants (*z* = −0.732, *p* = 0.232 and *z* = 0.704, *p* = 0.241, respectively). No comparisons were performed for Jewish and Muslim participants due to low case numbers. We have to reject our hypothesis regarding stronger effect strength differences of correlations of intelligence and religious beliefs and intelligence and religious behaviors in Protestant than in Catholic participants, as our results suggested quite the opposite.

##### *g*-loadings

**Figure 2:**
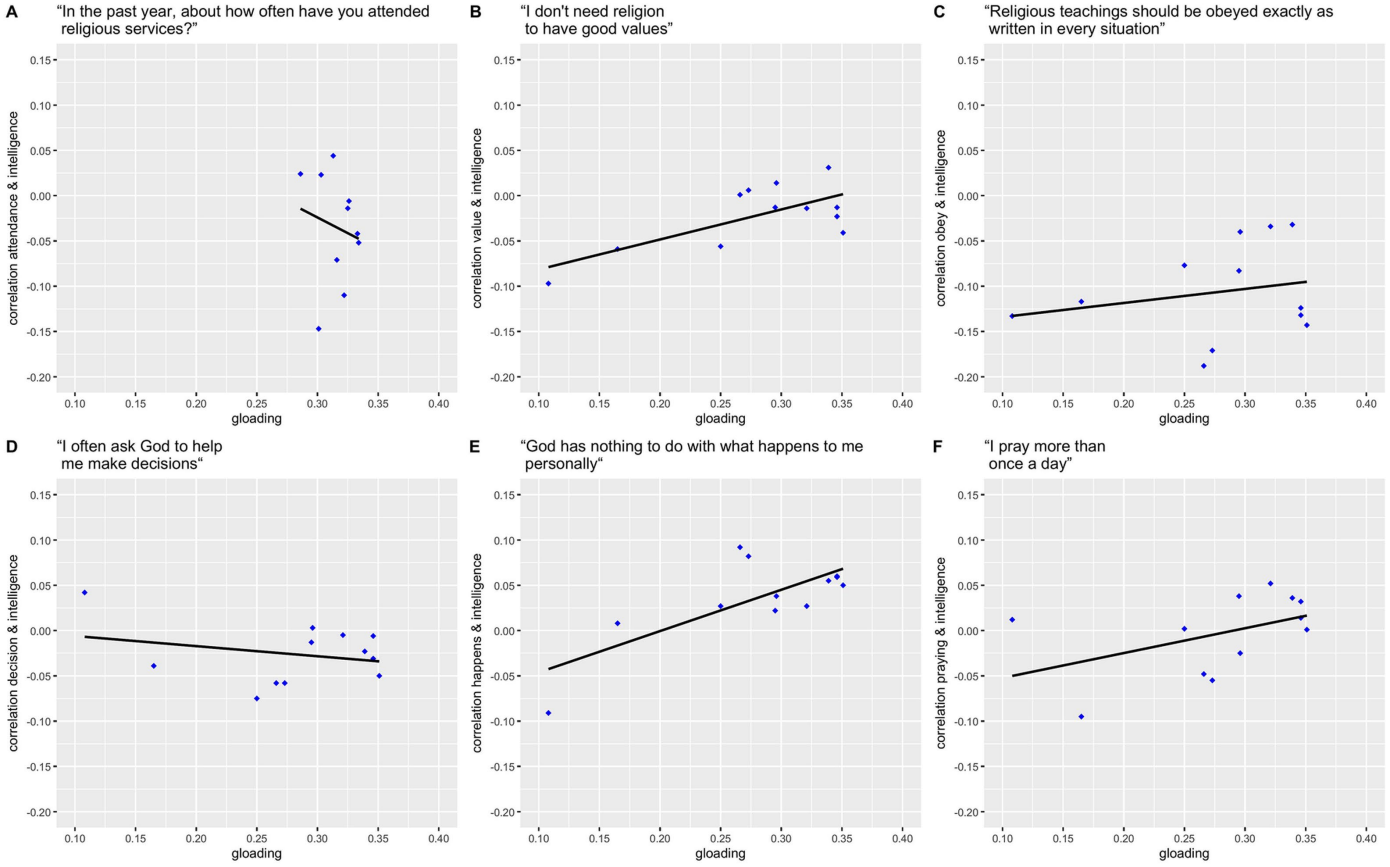
Regressions of associations of intelligence and religiosity associations on respective *g*-loadings of intelligence measures. **(A)** attendance = “In the past year, about how often have you attended religious services?” (not at all/ several times or less during the year/ about once a month/ two or three times a month/ about once a week/ more than once a week), **(B)** value = “I do not need religion to have good values” (0 = True; 1 = False), **(C)** obey = “Religious teachings should be obeyed exactly as written in every situation” (0 = False; 1 = True), **(D)** decision = “I often ask God to help me make decisions” (0 = False; 1 = True), **(E)** happens = “God has nothing to do with what happens to me personally” (0 = True; 1 = False), **(F)** praying = “I pray more than once a day”.

Regressing associations of the subtest scores and religiosity indices on *g*-loadings yielded no significant changes of cognitive ability correlations with the items “Religious teachings should be obeyed exactly as written in every situation” (*β* = 0.155, *p* = 0.485, *R*^2^ = −0.045, *η*^2^ = 0.05), “I often ask God to help me make decisions” (*β* = −0.112, *p* = 0.415, *R*^2^ = −0.026, *η*^2^ = 0.07), and “I pray more than once a day” (*β* = 0.274, *p* = 0.124, *R*^2^ = 0.142, *η*^2^ = 0.22). Associations of intelligence with “I do not need religion to have good values” (*β* = 0.332, *p* = 0.011, *R*^2^ = 0.441, *η*^2^ = 0.49) as well as of intelligence and “God has nothing to do with what happens to me personally” (*β* = 0.456, *p* = 0.006, *R*^2^ = 0.500, *η*^2^ = 0.54) were more pronounced for subtests with higher *g*-loadings (Figure 2), supporting our hypotheses regarding stronger associations of religious beliefs with tasks of higher *g*-loadings. Formal tests for differences in strength of (CAT-)ASVAB-subtests and religiosity measures associations are numerically detailed in Supplementary File S4 at https://osf.io/9zvre. Table 4 provides *g*-loadings of ASVAB and CAT_ASVAB subtests (in descending order) as well as their associations with respective religiosity measures.

### Discussion

In our primary data approach, we assessed intelligence and religiosity associations within two cohorts of the NLSY. As expected, a vast majority of intelligence and achievement measures were negatively correlated with religious beliefs in both cohorts. These results are consistent with our hypothesis regarding negative associations of intelligence and religious beliefs and conform to prior evidence (Dürlinger and Pietschnig, 2022). Furthermore, positive associations of religious beliefs and religious behaviors fit well to our expectations.

Formal comparisons within the 1997-cohort yielded significant differences between religious beliefs and intelligence associations and religious behaviors and intelligence associations for Catholic, but not for Protestant participants. This is surprising, because we considered religious behaviors to possess a lower status in Protestantism. We expected accordingly associations with intelligence to be less pronounced for religious behaviors than for religious beliefs in Protestant participants, but not in Catholic participants.

However, because of the relatively low status of religious behaviors in Protestantism, those who engage in religious behaviors may do so out of personal conviction rather than social pressure or conventions. For Protestants, engaging in religious behaviors may therefore be a better indicator for personal beliefs than for Catholics. This may explain the presently observed stronger associations of religious beliefs and intelligence than of religious behaviors and intelligence in Catholics, but not in Protestants.

Moreover, it is likely that praying having been used as an indicator of religious behaviors has contributed to these results. Praying “can be considered as a better proxy for the actual beliefs of a person than religious attendance, because it can be assumed to represent a consequence of an intrinsic motivation” (Dürlinger et al., 2023, p. 5). Moreover, praying has been found to correlate stronger with intelligence than religious attendance and has been used in the past as a measure of religious beliefs (Dürlinger et al., 2023). Nevertheless, praying was virtually unrelated to intelligence in Catholic and in Protestant participants, indicating that it is not equivalent to personal religious beliefs and might be motivated by further means other than personal beliefs.

Moreover, neither associations of intelligence and religious beliefs nor associations of intelligence and religious behaviors differed in Catholic vs. Protestant participants. We therefore conclude that praying is of similar meaning when it comes to relations with intelligence in both denominations thus suggesting that stronger negative associations of intelligence and religious beliefs compared to behaviors are not limited to Protestantism (but see Zuckerman et al., 2013, for a contrasting account).

We found indications for more pronounced intelligence and religiosity associations for intelligence-subtests with higher *g*-loadings, indicating less religiosity in individuals with higher general intelligence. These results are consistent with previous findings of individual-level data analyses (Dutton and Kirkegaard, 2021) and may be interpreted as tentative evidence for intelligence influencing religiosity causally rather than the other way around. In this vein, this proposed causal mechanisms might be suitable to explain negative intelligence and religiosity associations. First, frequently observed consistent positive associations of analytic thinking styles with several different intelligence domains like reasoning and abstract thinking (Alaybek et al., 2021) clearly show that analytic thinking styles are related with psychometric *g*. However, a preference for analytic cognitive styles has been shown to be negatively associated with religiosity (Gervais and Norenzayan, 2012), thus suggesting a negative link between *g* and religiosity and thus conforming to our results.

Second, positive correlations of *g*-loadings and the strength of intelligence and religiosity associations conform to the idea of a functional equivalence of intelligence and religiosity. The presently observed positive, albeit weak, associations of self-control and various cognitive abilities, such as vocabulary comprehension, working memory or spatial orientation (Yeh et al., 2021) is consistent with the idea that general intelligence helps in fulfilling similar needs and desires as religiosity. Evidence for negative correlations of a sense of compensatory control with abstract and verbal reasoning further support this idea (Wood and Englert, 2009; Prenda and Lachman, 2001).

Third, assumptions that individuals that score lower on intelligence tests are more likely to adopt belief systems from their (religious) surroundings than people of higher general intelligence are supported by positive associations of non-conformity and general intelligence (Millet and Dewitte, 2007), abstract reasoning and verbal reasoning (Wood and Englert, 2009).

In all, associations of general intelligence as well as religiosity with (i) analytic thinking styles, (ii) psychological needs and desires, as well as (iii) (non-)conformity, indicate a causal pathway of general intelligence on religiosity.

## Study 2

### Materials and methods

#### Literature search

**Figure 3:**
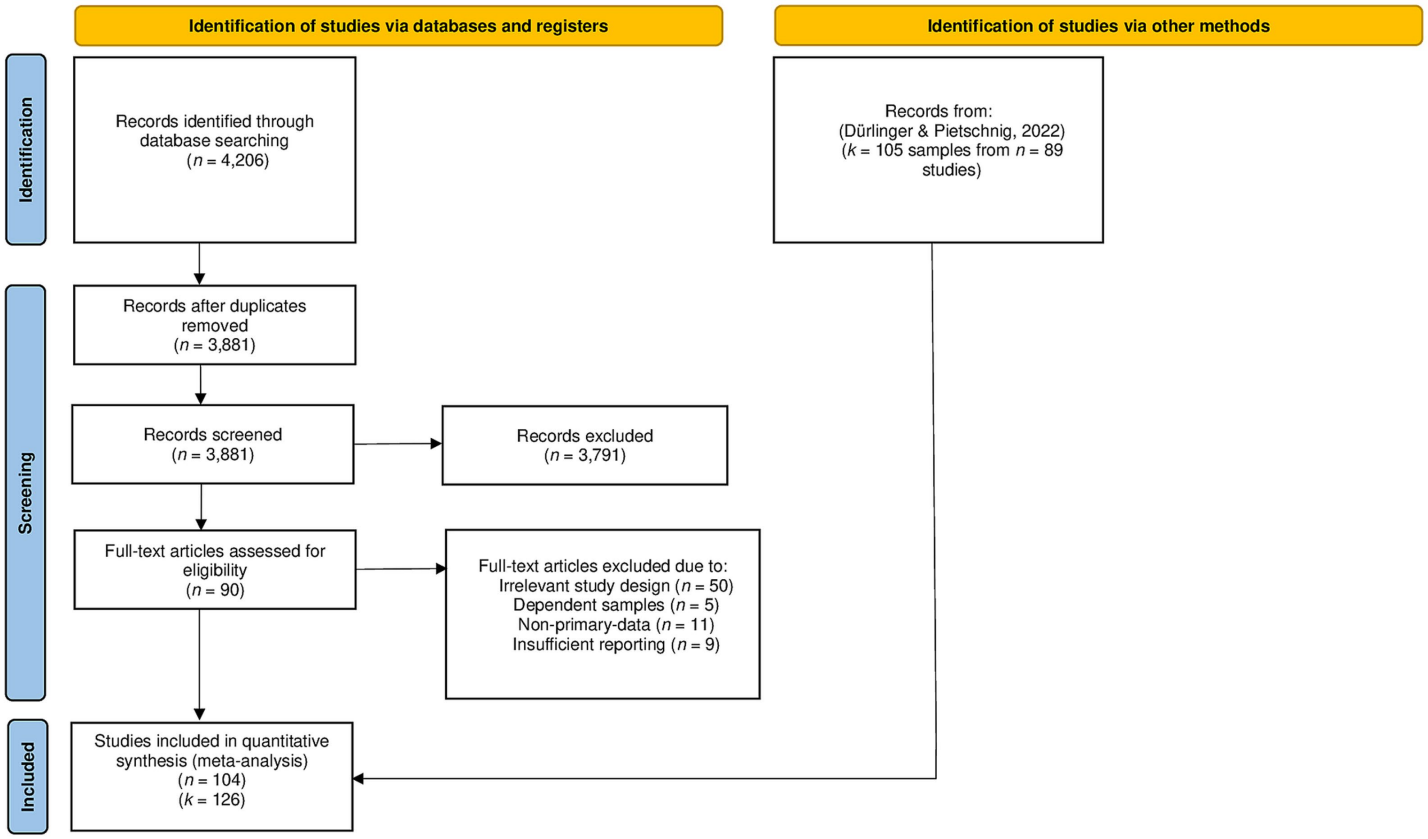
Flow-chart of study inclusion.

For our meta-analytic approach, we initially obtained studies from previous meta-analyses on this topic (Dürlinger and Pietschnig, 2022; Zuckerman et al., 2020). Subsequently, we searched for potentially relevant studies in five databases that index published items (ISI Web of Knowledge, Scopus, Pubmed, PsycINFO, Google Scholar) as well as in the Open Access Theses and Dissertations database^1^ that indexes items from the grey literature. In addition, volumes from the “Archive for the Psychology of Religion,” the “Journal for the Scientific Study of Religion,” and “Review of Religious Research” in years not indexed by PsycINFO were manually screened. To identify titles and abstracts of possibly includable studies, the following search string was used: (“intelligence quotient” OR IQ OR intelligence OR “cognitive ability”) AND (religious OR spirituality OR religiosity OR “religious beliefs”). In total, we screened titles and abstracts of 3,881 articles (see Figure 3 for a flow-chart) and subsequently assessed full-texts of 87 studies. The data collection procedure for the meta-analysis was carried out in July and August 2024.

#### Inclusion criteria

To be eligible for inclusion in the meta-analysis, studies had to meet the following criteria: First, a correlation of psychometrically assessed intelligence or academic achievement measures and religiosity had to be reported or alternatively the necessary statistics to compute such a correlation coefficient had to be available. Second, we only included samples comprising healthy participants. Third, included data had to be independent of one another. In cases of data dependencies, we preferred (i) larger and (ii) more recently published records. Finally, studies had to be published either in English or in German.

#### Coding

Coding of studies into categories (type of religiosity measure: beliefs vs. behaviors vs. mixed; publication status: published vs. unpublished; sample type: pre-college vs. college vs. general population), recording of other relevant variables (publication year, effect sizes, their associated *p*-values, type of intelligence measure and number of intelligence subtests) as well as sample characteristics (sample size, percentage of men within samples) was conducted twice by the same experienced researcher (FD). Inconsistencies in coding were resolved by discussion with an independent researcher (JP). Inclusion of correlations of religiosity with psychometric intelligence tests was preferred over those with achievement measures (e.g., GPA).

#### Final sample

For our meta-analytical approach, we included *k* = 126 independent samples from *n* = 104 studies, conducted between 1928 and 2024 (see https://osf.io/b7xye for detailed study characteristics). A majority of studies was published (*k* = 104) and used psychometric tests to assess intelligence (*k* = 114).

#### Analyses

For the meta-analytical approach, we used random-effects models to estimate summary effects of intelligence and religiosity correlations. For the main analyses, associations of intelligence with measures of religious beliefs were preferred for inclusion over associations of intelligence with measures of religious behaviors. If a study provided more than one effect size for the same association (due to having used either several intelligence tests or several measures of religious beliefs), correlations were averaged using *z-*transformed values.

In addition, we conducted two exploratory multilevel meta-analyses, thus accounting for data dependencies whilst maintaining the maximum of possibly includable effect sizes. In the first model, studies providing associations of religiosity with more than one measure of intelligence were synthesized. In the second model, studies providing associations of intelligence with more than one measure of religiosity were included. By integrating a third level in the structure of the random effects model, data-dependency (correlations are derived from the same samples) can be taken into account. In our three-level meta-analyses, effect sizes (level 1) are nested in studies (level 2) and then pooled to an overall effect size (level 3). However, we report all other results based on two-level meta-analyses because results from two- and multilevel-modeling were virtually identical and interpretations of moderator analyses and specification curve analyses are more straightforward (see below).

Subgroup-analyses were conducted by means of mixed-effects models (i.e., effect size estimates for intelligence and religious beliefs or behaviors associations of each subgroup were based on random-effects models; between-subgroup comparisons were based on fixed-effect models). To examine potentially moderating effects of test *g*-loadedness, we classified correlations within primary studies based on the expectable *g*-loadings of the used ability measure. We used the number of subscales that cognitive test scores were based on for classification, following prior approaches (e.g., Gignac and Bates, 2017): Test scores based on 1, 2, 3–8, and 9+ subscales were considered to represent “poor,” “fair,” “good,” and “excellent” *g*-loadedness, respectively.

#### Multiverse and specification curve analyses

Primary and meta-analytical study outcomes alike may be affected by several (reasonable) decisions that need to be made by the authors of a respective study during conceptualization, assessment, and analysis of a given study. In the meta-analytical context, particular importance can be attributed to choices that authors have to make about i) which data is going to be analyzed (“Which”-factors; e.g., choices about inclusion criteria or subgroup analyses) and ii) how to analyze the data (“How”-factors; e.g., choices about the use of analytical approaches). Different choices may lead to a large number of different (reasonable) ways to analyze a specific set of data pertaining to the very same research question. Multiverse and specification curve analyses allow for assessing the generality of a given effect by examining the influence of different choices that have been made on the meta-analytical outcome (Voracek et al., 2019).

In this vein, we included four “Which” factors (i.e., which data were meta-analyzed) and two “How” factors (i.e., how data were meta-analyzed) (see Supplementary File S5 at https://osf.io/qgfcy/files/ackxn for further details).

#### Inferential test of the specification-curve analysis

The descriptive meta-analytic specification curve is indicative of the robustness of an effect. However, we applied a parametric bootstrap approach (see Voracek et al., 2019) to formally test whether the null hypothesis of no effect can be rejected (see Supplementary File S5).

#### Combinatorial meta-analysis

Combinatorial meta-analyses follow a similar idea as specification curve analyses, but account for the fact that not all potentially moderating variables may be known before conducting a meta-analysis, thus rendering specification curve analyses potentially insufficient to detect influences of unobserved heterogeneity. Therefore, in this method all 2^*k* − 1^ possible ways to synthesize the available meta-analytical data are deemed to be informative about the robustness of a meta-analytic effect whilst allowing assessment of systematic influences of influential studies (see Supplementary File S5).

#### Dissemination bias

We applied several standard as well as more novel methods to assess potential dissemination bias (excepting *p*-uniform*, only published studies were included in these analyses; for a detailed description of these methods, refer to Siegel et al., 2022). First, funnel-plots were visually inspected for indications of asymmetry. Second, parametric (Sterne and Egger, 2005) and non-parametric methods (Begg and Mazumdar, 1994) were applied to formally examine possible funnel plot asymmetry. Third, we used the Trim-and-Fill method (Duval and Tweedie, 2000) to estimate the number of missing studies in order for the funnel plot to become symmetric. Of note, resulting adjusted effect reestimations should be interpreted in terms of a sensitivity analysis. Fourth, we examined a potential excess of significant studies (Ioannidis and Trikalinos, 2007). To do so, we obtained the average power of the primary studies to detect the observed meta-analytical summary effect. These power estimates are then used to calculate expected frequencies of significant studies, which are in turn compared with the number of observed significant studies. Finally, we used three *p*-value-based methods for bias assessments (*p*-curve: Simonsohn et al., 2014; *p*-uniform: van Assen et al., 2015; *p*-uniform*: van Assen et al., 2015).

### Results

**Figure 4:**
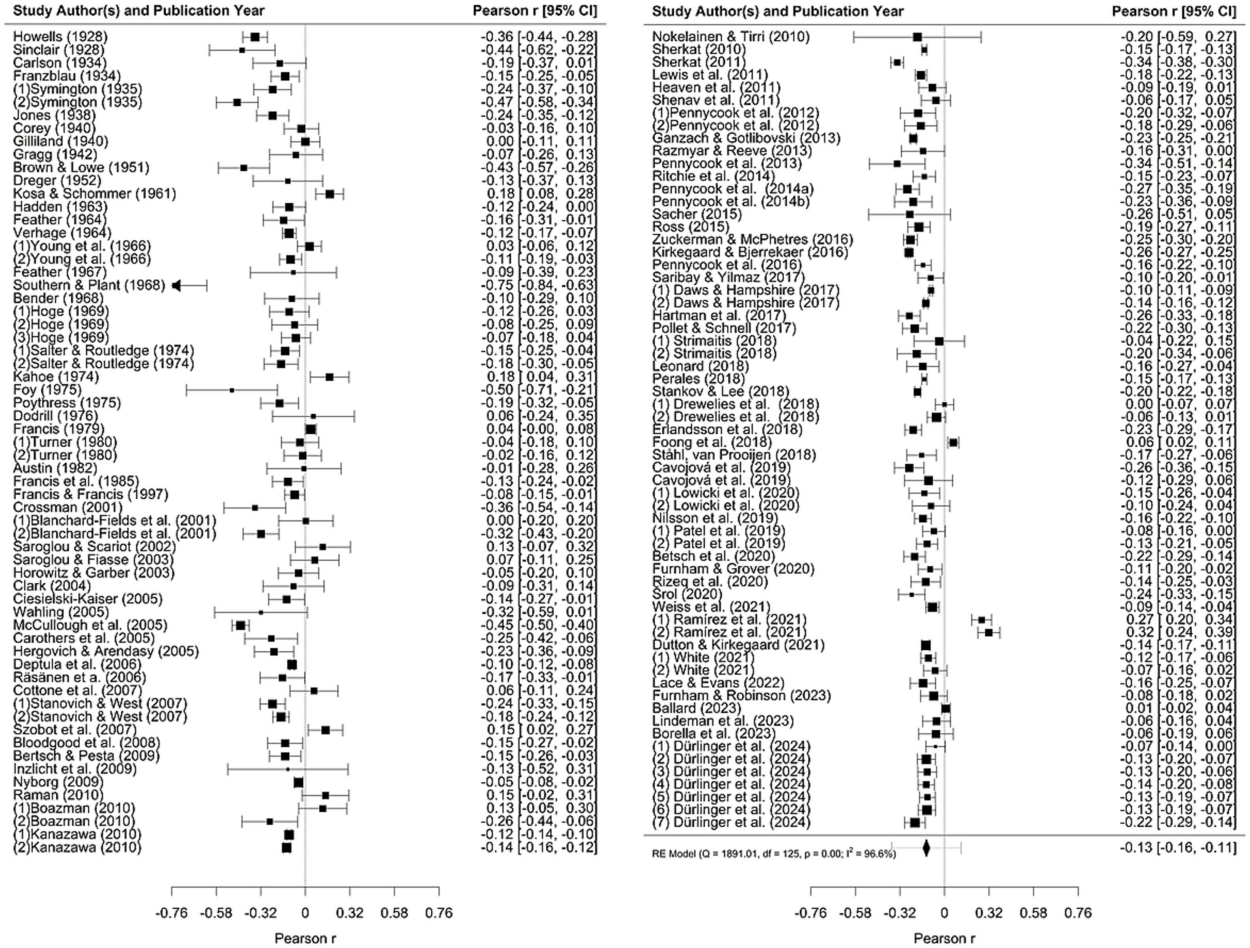
Rainforest plot for associations of intelligence measures with religiosity. Overall effect size calculations are based on random-effects models; the diamond represents the summary effect size; length of confidence intervals varies according to relative study weights within the analysis.

Meta-analytical examinations based on random-effects models yielded an overall effect of *r* = −0.14 [*p* < 0.001; 95% CI (−0.16, −0.11)]. A forest plot of our main analysis is provided in Figure 4. Substantial heterogeneity was observed (*Q* = 1891.01, *p* < 0.001, *I*^2^ = 96.57%, *τ*^2^ = 0.017, SE = 0.003), thus suggesting an influence of unobserved heterogeneity because of moderator variables. Outlier analyses by means of influence diagnostics (standardized residuals, DFFITS values, Cook’s distances, covariate ratios, leave-one-out values for heterogeneity test statistics, hat values, weights) revealed four leverage points. However, we report results based on all available data below because omitting the leverage points from analyses yielded virtually identical results (see Supplementary File S6 at https://osf.io/qgfcy/files/bvs3f).

Results of our subgroup analyses indicated that differences in intelligence and religiosity associations according to the *g*-loadedness of the used intelligence measures conformed to the expected direction, showing (barely non-significant) smaller effects for studies with lower *g*-loadedness (i.e., lower numbers of intelligence subscales; *Q* = 3.648, df = 1; *p* = 0.06). In a similar vein, associations of intelligence with measures of religious beliefs were more pronounced than those with religious behaviors. However, effect size differences did not reach nominal significance (*Q* = 0.989, df = 1; *p* = 0. 320). In total, our findings are consistent with the expected negative associations of intelligence with religious beliefs as well as with religious behaviors. Subgroup-specific effect estimates did not reach nominal significance, most likely due to the rather small number of studies that assessed intelligence with more than one subtest (*k* = 15 fair, *k* = 17 good, and *k* = 11 excellent *g*-loadedness). Analogously, the number of studies assessing religious behaviors is comparatively small (69% beliefs vs. 11% behaviors), resulting in large confidence intervals of the corresponding summary effects. The results still support our expectations regarding stronger relations of intelligence with religious beliefs than with religious behavior. Descriptive statistics of summary effects of the subgroup analyses are provided in Table 7.

**Table 7:** Random-effects estimates of all data and according to intelligence subtest numbers.

	Summary effect (*r*)	SE	95% CI	*Q*	*I*^2^
Overall (*k* = 126)	−0.135^***^	0.013	[−0.160, −0.110]	1891.0057^***^	96.57%
Number of intelligence subtests					
Poor *g*-loadedness (*k* = 43)	−0.123^***^	0.021	[−0.164, −0.082]	483.4058^***^	96.26%
Fair *g*-loadedness (*k* = 15)	−0.129^***^	0.030	[−0.186, −0.070]	47.3646^***^	73.52%
Good *g*-loadedness (*k* = 17)	−0.170^***^	0.021	[−0.211, −0.130]	70.9138^***^	83.35%
Excellent *g*-loadedness (*k* = 11)	−0.195^***^	0.050	[−0.286, −0.100]	192.9840^***^	98.77%
Religiosity assessment					
Beliefs (*k* = 87)	−0.148^***^	0.017	[−0.180, −0.116]	1426.9262^***^	96.53%
Behaviors (*k* = 14)	−0.084^**^	0.034	[−0.150, −0.017]	98.2424^***^	96.84%
Mixed (*k* = 25)	−0.126^***^	0.023	[−0.170, −0.080]	226.1657^***^	91.63, 92.32%

SE, standard error; 95% CI, 95% lower and upper bound of 95% confidence interval; *Q*, Cochran’s *Q* test statistic for heterogeneity; *I*^2^, ratio between true heterogeneity and total observed variation. ^**^p < 0.01 and ^***^p < 0.001.

#### Multiverse and specification curve analyses

**Figure 5:**
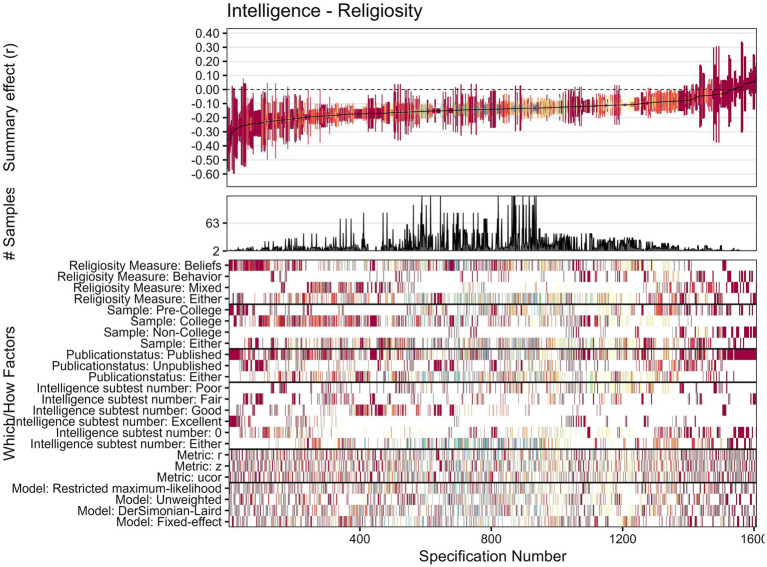
Descriptive meta-analytic specification-curve plot. Specifications’ summary effects with their associated 95% confidence intervals are illustrated sorted by magnitude. Directly below is the number of samples contained in the corresponding meta-analytic specification displayed, and below that one can see the combination of which and how factors constituting each specification. Colors in this pattern indicate the number of samples included in the corresponding specification. Warm colors (red, orange, yellow) indicate that very few samples are included in the respective effect estimation, whereas cool colors (blue, green, violet) indicate a larger number of samples in a given effect estimation. The combinations of which and how factors constituting each specification are displayed in the bottom part. Corresponding summary effects are shown in the top part.

**Figure 6:**
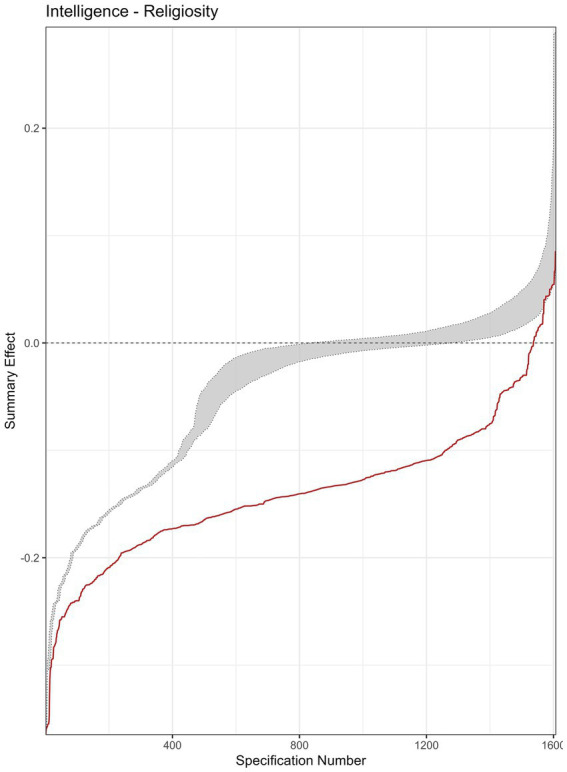
Inferential meta-analytic specification plot. The specification curve (red) of the effect strength-sorted observed meta-analytic summary effects for all specifications is compared to the under-the-null scenario of a possible zero effect (grey).

**Figure 7:**
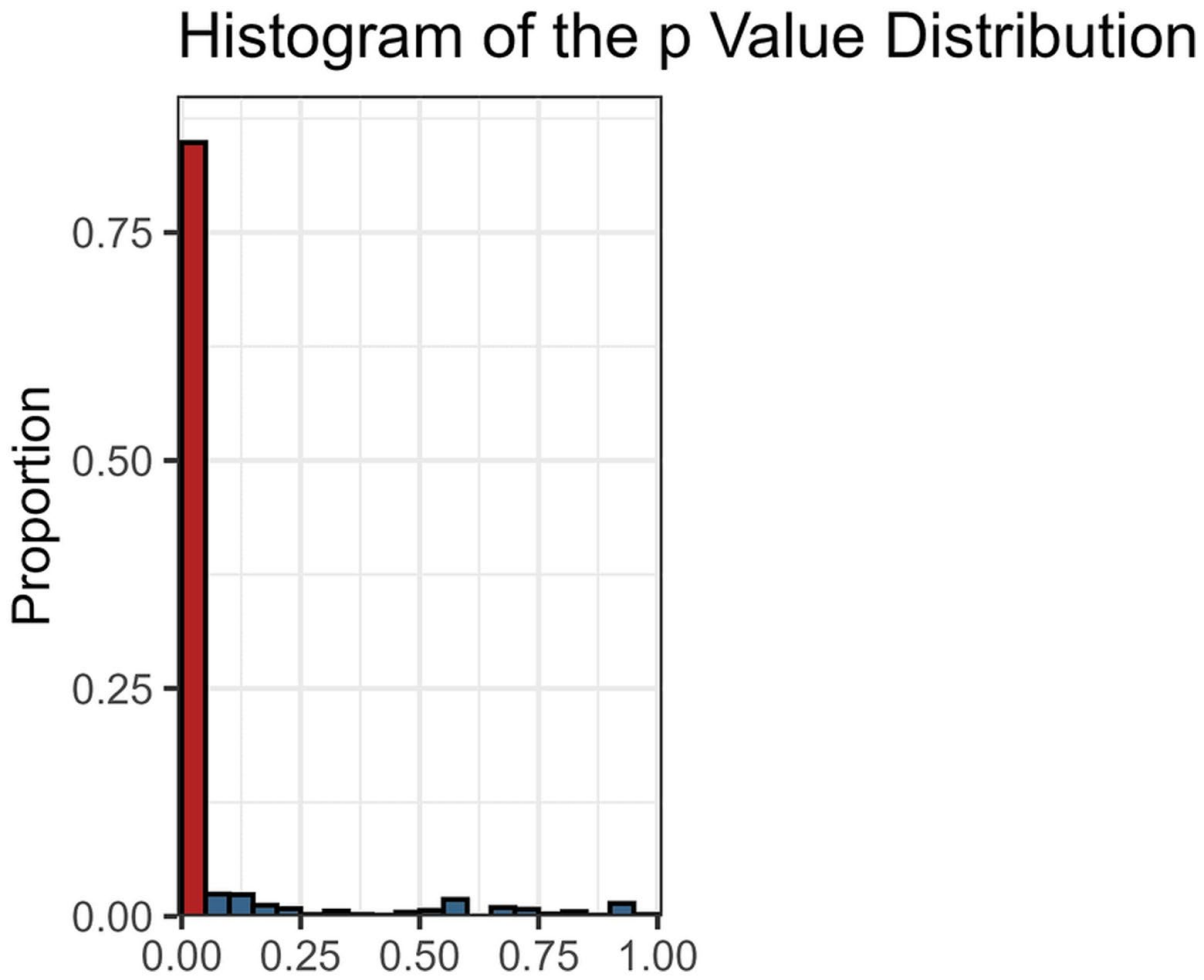
Histograms of *p*-values for all meta-analytic specifications. The proportion of nominally significant values (*p* < 0.05) is highlighted in red.

**Figure 8:**
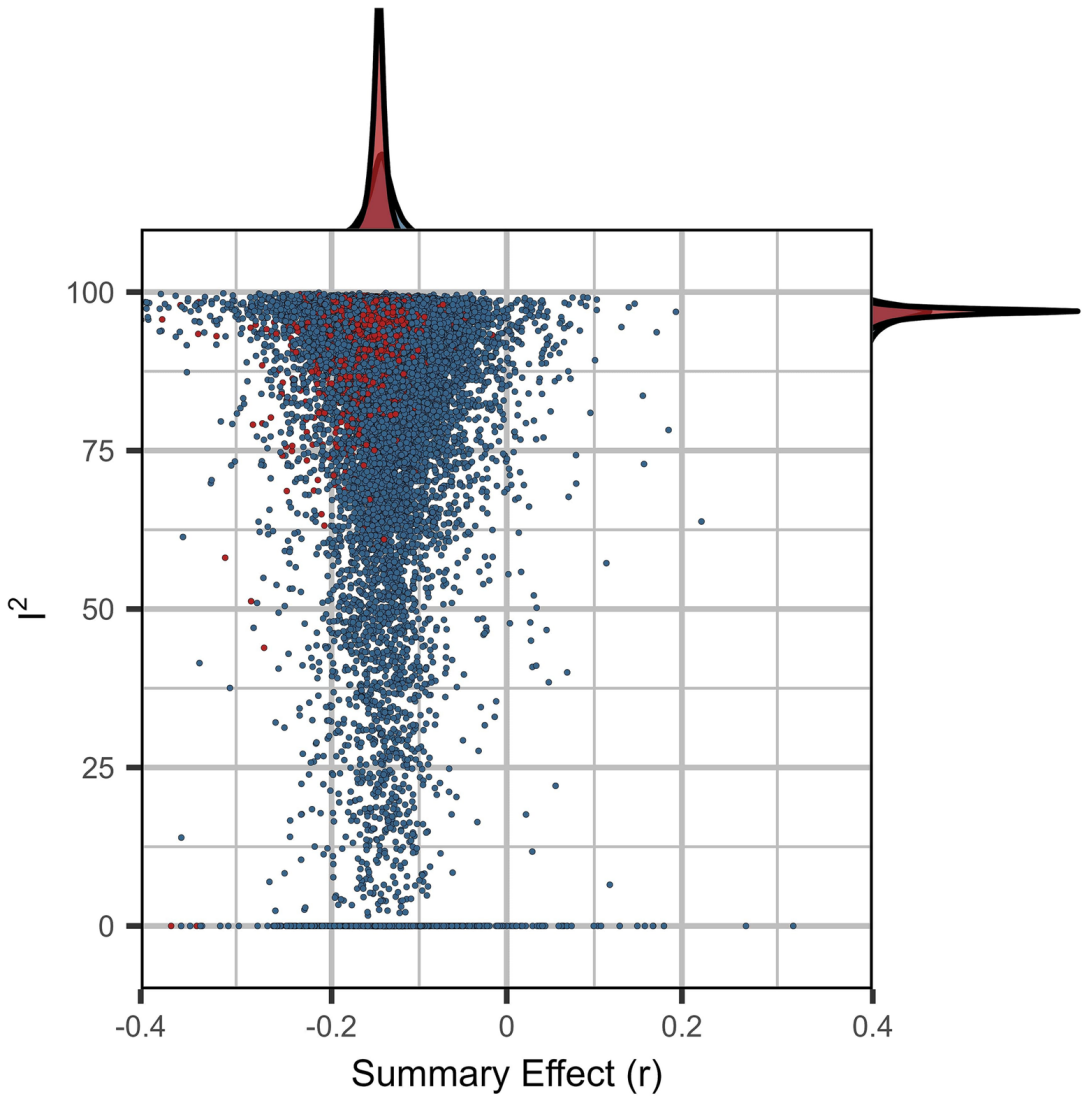
GOSH plot for combinatorial meta-analysis. Each dot represents the summary effect of a random subset of studies. A random sample of 100,000 different subsets is depicted; subset estimations including at least one of the leverage points are highlighted in red.

The descriptive meta-analytic specification-curve plot is provided in Figure 5. In all, 1,608 specifications comprised more than a single study, yielding 1,363 (85%) nominally significant (*p* < 0.05) negative and 9 (<1%) significant positive summary effects. The observed results clearly deviate from the under-the-null scenario of an underlying nil effect (Figure 6), indicating a robust negative association. The histogram of the *p*-value distribution for the summary effect of the various meta-analytic specifications is provided in Figure 7. There is an obvious excess of *p*-values smaller than 0.05, thus further corroborating the robustness of negative intelligence and religiosity associations. The combinatorial meta-analyses are visualized in the GOSH-plot (graphical display of study heterogeneity) in Figure 8.

#### Dissemination bias

**Figure 9:**
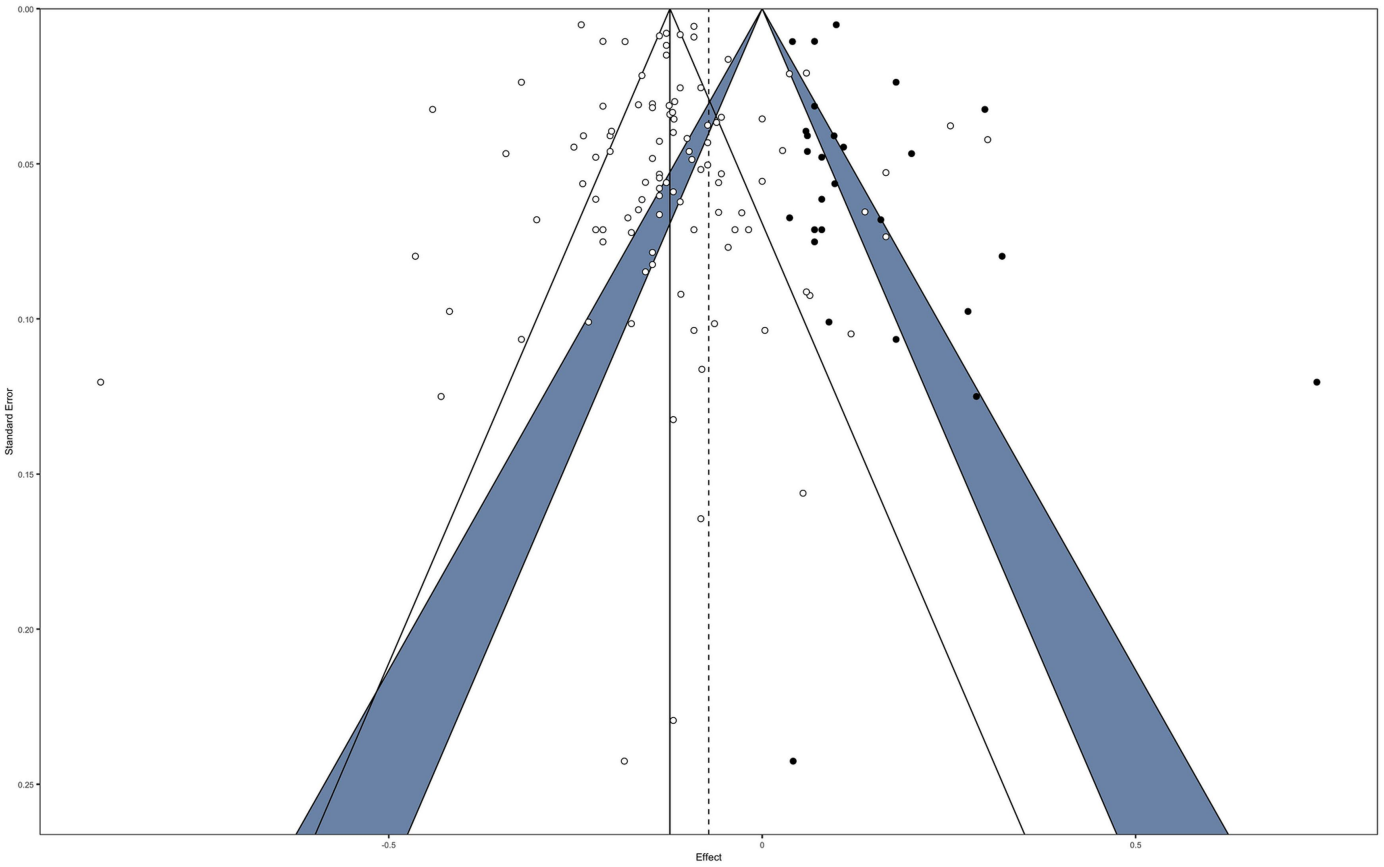
Contour-enhanced funnel plot of published effect sizes (*k* = 88). The dashed line represents the summary effect estimate; the vertical line represents the null effect; confidence lines delimit non-significance of effect sizes within (*p*-values within areas: white >0.05, blue <0.05).

Visual funnel plot inspection (Figure 9) shows some signs of asymmetry. Neither Sterne and Egger’s regression test (*Z* = −0.81, *p* = 0.42) nor the rank correlation method (*τ* = −0.109, *p* = 0.10) yielded evidence for funnel plot asymmetry. The Trim-and-Fill method estimated 27 studies missing on the right side. Trim-and-fill based adjusted random-effects calculations yielded a summary effect of *r* = −0.08 [*p* < 0.001; 95% CI (−0.11; −0.05)], thus indicating some evidence for confounding bias.

In order to test for excessive significance, the average power based on the observed summary effect was obtained (within-study average power = 65%). The resulting number of expected significant studies in the hypothesis-conforming direction was 82. We observed less studies with a significant outcome than would have been expected based on our power calculations. Therefore, the test of excessive significance showed no indication of bias.

**Figure 10:**
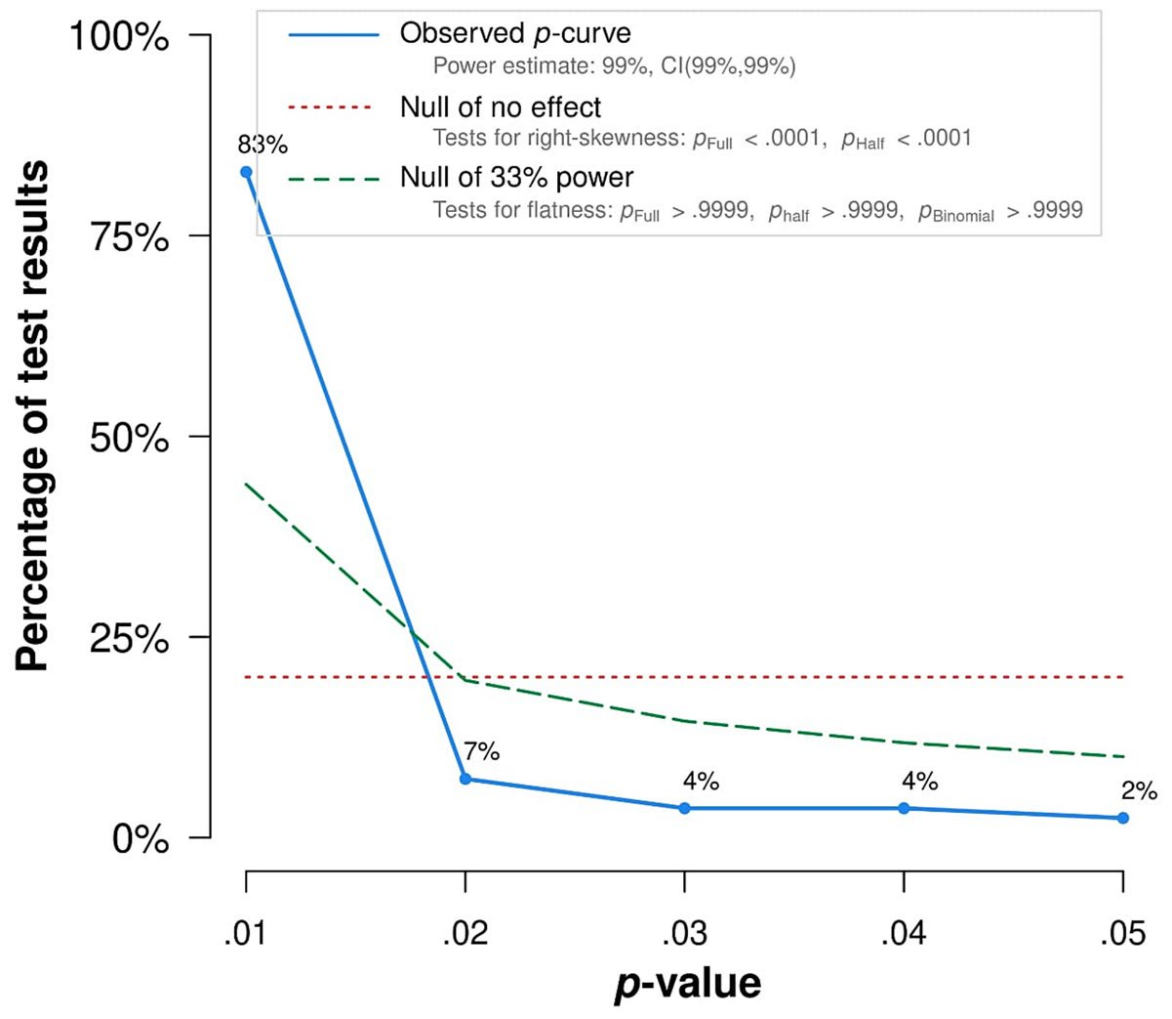
*p*-curve. Distribution of significant (*α* < 0.05) *p*-values of published findings. The observed *p*-curve includes 82 statistically significant (*p* < 0.05) results, of which 76 are *p* < 0.025. There were 44 additional results entered but excluded from *p*-curve because they were *p* > 0.05.

We conducted *p*-curve and *p*-uniform analyses with 64 published significant study effects. The resulting *p*-curve (Figure 10) is right-skewed with significantly larger numbers of small than large *p*-values. This indicates that there is sufficient empirical evidence of the present body of research to assume a non-nil effect and that the extent of confounding *p*-hacking is negligible. *p*-curve-based summary effect estimation yielded *r* = −0.17, which is broadly in line with the results from our standard random-effects analyses. In the *p*-uniform analyses, the distribution of conditional *p*-values of the fixed-effect estimate did not differ significantly from the uniform distribution (*p* = 0.27). This indicates no evidence for dissemination bias. *p*-uniform-based effect estimation yielded *r* = −0.16 [95%CI (−0.18; −0.15), *p* < 0.001], thus conforming to *p-*curve and standard estimation.

Similar to the *p*-curve and *p*-uniform estimates, *p*-uniform* yielded an estimate of *r* = −0.17. In all, our dissemination bias detection methods indicated no plausible evidence for confounding bias.

#### Multilevel meta-analyses

In some cases, studies reported more than one effect size of interest (associations of more than one intelligence measure with religiosity or associations of more than one religiosity measure with intelligence; see Supplementary File S7 at https://osf.io/qgfcy/files/rg96y). Therefore, we conducted two exploratory three-level meta-analyses, fitting effect sizes (level 1) in studies (level 2), pooled to an overall effect size (level 3).

In the first model, associations of more than one intelligence measure with religiosity were included (*k* = 164 effect sizes). Between-studies variances (*σ* = 0.012, *I*^2^ = 71.14, *k* = 121) were larger than within-studies variances (*σ* = 0.005, *I*^2^ = 25.91, *k* = 146). The model yielded an overall effect of *r* = −0.134 (*p* < 0.001). Fitting an identical model under the assumption that all effect sizes are independent (i.e., setting between-studies variance to zero, thus removing level 3) yielded a virtually identical summary effect (*r* = −0.135, *p* < 0.001), although the full (three-level) model showed a better model fit (AIC = 23.65, BIC = 32.93) than the reduced model (AIC = 28.01, BIC = 34.20). The likelihood ratio test comparing both models was significant (*p* = 0.012), indicating that the inclusion of between-study variance was justified.

In the second model, associations of more than one religiosity measure with intelligence were included (*k* = 182 effect sizes). Between-studies variances (*σ* = 0.009, *I*^2^ = 51.05, *k* = 120) were slightly larger than within-studies variances (*σ* = 0.008, *I*^2^ = 44.73, *k* = 182). The model yielded an overall effect of *r* = −0.128 (*p* < 0.001). Fitting an identical model under the assumption that all effect sizes are independent (i.e., setting between-studies variances to zero, thus removing level 3) yielded slightly smaller summary effects (*r* = −0.120, *p* < 0.001). Here, the reduced model (AIC = −177.51, BIC = −171.12) showed a better model fit than the full (three-level) model (AIC = −185.43, BIC = −175.83). The likelihood ratio test comparing both models was significant (*p* < 0.001). Although the three-level model does not provide a better model fit, the data-generating process is still better represented because the importance of the cluster variable is clear (multiple effect sizes reported in one study are not independent). However, this dependence can be neglected due to the similarity of the summary effects.

## Discussion

In the present meta-analysis, we show stronger intelligence and religiosity associations for measures with higher *g*-loadings. This indicates that the negative intelligence and religiosity associations are on *g*, rather than on specific cognitive abilities. Our results are consistent with our findings of individual-level data analyses and are supported by previous findings (Dutton and Kirkegaard, 2021; but see Dutton et al., 2020 for contrasting findings based on group-level data).

Here, we used the number of intelligence subtests as an indicator of *g*-loadedness of the intelligence measure (Gignac and Bates, 2017) to examine if intelligence and religiosity associations are differentiated according to intelligence measure *g*-loadedness.

The strength of intelligence and religiosity associations steadily increased from poor, to fair, good, and excellent *g*-loadedness, thus conforming to our evidence from individual-level data in Study 1.

In terms of the stability of the intelligence and religiosity link, results of several standard and modern methods to detect dissemination bias convergently indicated negligible effects of dissemination bias. Our random-effects models yielded virtually identical summary effects as previous meta-analyses (e.g., Zuckerman et al., 2013; Dürlinger and Pietschnig, 2022) with multilevel models further supporting these results. Moreover, all data subsets yielded negative summary effects and the vast majority of reasonable specifications (including the number of intelligence tests in primary studies) showed that the negative intelligence and religiosity association generalizes across potential moderator variables and remains directionally consistent even when accounting for intelligent subtest number.

### General discussion

In the present study, we examined the role of test *g*-loadedness and religious denominations for the intelligence and religiosity association. In two independent investigations, we observed consistently negative associations that appeared to be stronger for higher *g*-loaded tasks. Associations of intelligence and religious beliefs were more pronounced than associations of intelligence and religious behaviors in Catholic, but not in Protestant participants. Our results present several points of interest, as we discuss below.

First, our results indicate stronger religiosity associations for intelligence tests with larger *g*-loadings. Arguably, our primary data analyses only provide tentative support for this idea, because associations of intelligence and religiosity were not differentiated according to *g*-loadings in the NLSY79-cohort and only for two of five indicators of religiosity in the NLSY97-cohort. However, results of our meta-analytical investigations corroborate this interpretation, yielding findings with a steady increase of effect strengths in studies with more comprehensive intelligence assessments. This means that individuals of higher general intelligence are less likely to uphold religious worldviews and that specific cognitive skills are weaker predictors of religiosity than general intelligence.

Interestingly, there are isolated empirical accounts of fundamental religious beliefs limiting the development of verbal abilities (Sherkat, 2010) and thus suggesting that religiosity may affect intelligence. However, although our correlational data does not allow drawing causal inferences, more pronounced intelligence and religiosity associations for higher *g*-loaded tasks tentatively support the idea that intelligence affects religiosity instead the other way around. Non-religious people being of higher general intelligence than religious people conform to the idea of a functional equivalence of intelligence and religiosity. For instance, the need for a consistent worldview might be fulfilled by more intelligent individuals through explanations that follow a scientific approach whereas less intelligent individuals may resort to less complex explanations provided by religiosity (Nyborg, 2009).

Moreover, assumptions that effects of intelligence on religiosity are mediated by a tendency of conforming to beliefs systems from religious surroundings are supported by positive relations of the intelligence and religiosity link with *g*. It is general intelligence, not any specific cognitive skill, that fosters non-conformity (Millet and Dewitte, 2007) and non-conformity that should encourage departing from religion in religious societies (Zuckerman et al., 2013).

Stronger religiosity associations for intelligence tests with larger *g*-loadings also conform to the idea of analytic thinking styles acting as a mediator of intelligence and religiosity associations. Prior evidence suggested that individuals with higher general intelligence tend to prefer analytic thinking styles (Alaybek et al., 2021) and that analytic thinking styles are associated with less religiosity (Gervais and Norenzayan, 2012). In fact, analytic thinking styles have been demonstrated to partially mediate associations of intelligence and religiosity (Dürlinger and Pietschnig, 2022). Our results show that people of higher general intelligence are less likely to be religious, thus supporting the mediating role of analytic thinking styles.

Assumptions of a causality of intelligence on religiosity are further supported by evidence that intelligence develops earlier than religiosity. Intelligence can be measured reliably early in life (Kanazawa, 2010) and remains largely stable throughout adulthood (Larsen et al., 2008) whilst religiosity cannot be measured reliably that early and is more malleable than intelligence (O’Connor et al., 2002). However, we note that due to the correlative nature of our investigation, we were unable to formally establish causal effects and that alternative explanations cannot be completely ruled out here.

Second, we found more pronounced associations of intelligence and religious beliefs than of intelligence and religious behaviors in Catholic, but not in Protestant participants in the NLSY97-cohort. We expected these differences to be larger in Protestants than in Catholics because we considered religious behaviors to be more important in Catholic dogmas. In accordance with that, it has been hypothesized “that the emphasis on beliefs as the intrinsic component of religiosity (and, as such, the component with stronger negative relation to intelligence) might be an attribute of American Protestant religion, and may be less true of Judaism and Catholicism” (Zuckerman et al., 2013; p. 22), although no empirical evidence for this idea had been provided thus far.

Our findings indicate that in the presently investigated US-American sample, Catholics do not place more emphasis on religious behaviors than Protestants. Therefore, religious behaviors might be considered as less indicative for personal beliefs in Catholics compared to Protestants, but rather represent ritualized automatized behaviors, conducted without much conscious effort. Notably, while religious behaviors were only measured via praying frequencies in the NLSY97-cohort, associations of intelligence and religious attendance were stronger for Protestants than for Catholics in the NLSY79-cohort, while attendance in religious services was similarly widespread among Protestants and Catholics with 13% of Catholics and 14% of Protestants reporting no attendance at all.

Therefore, praying and attendance of religious services do not appear to differ between Protestants and Catholics in terms of their importance. It needs to be noted that in contrast to the rather homogeneous group of Catholic Christians, the protestants in our sample varied considerably in terms of their denominations, with Baptism (*N* = 3,211), Methodism (*N* = 850), Lutheranism (*N* = 601), Presbyterianism (*N* = 277), and Episcopalianism (*N* = 171) being the most common ones. In the present investigation, we analyzed their data in a common group because an emphasis on personal beliefs as the intrinsic component of religiosity should be an attribute of all these denominations. Ideas of God forgiving sinners “on the basis of faith only, not on the basis of any human works or human merit or human righteousness” (Campbell, 1996, p. 120) are common propositions in these denominations. In Protestant denominations, the Bible is considered the ultimate source of Christian teaching in Protestantism and other forms of clerical institutions are of a lower status compared to other branches of Christianity (Campbell, 1996).

However, Protestant affiliations place emphasis on religious behaviors too. For instance, a distinguishing feature of Baptism (the second largest denomination in the US after Catholicism) is the baptizing of only professing Christian believers (Yarbrough and Kuykendall, 2021), whilst Methodists put special emphasis on social engagement (Maddox, 1994), Lutherans are committed to social justice and humanitarian concerns (Willer, 2017) and Episcopalianism is heavily influenced by Catholic tradition, exhibiting very similar rituals and worships (Sirota, 2024).

Associations of intelligence and religious behaviors (indicated by attendance at religious services) were not differentiated according to *g*-loadings in individual-level data. Possibly, this may be due to religious behaviors representing a rather noisy measure of personal beliefs, especially in the NLSY79 cohort where only frequencies of religious attendance were assessed. However, we found the expected more pronounced associations with intelligence for religious beliefs than for religious behaviors in our meta-analysis, thus supporting prior accounts (Zuckerman et al., 2013; Dürlinger and Pietschnig, 2022). Our findings of larger intelligence and religious beliefs than intelligence and praying associations in the NLSY97 cohort are consistent with our meta-analytical observations.

Although religious beliefs and religious behaviors associations yielded the expected positive direction, associations were mostly weak in strength excepting large associations of praying behavior with asking God to help in making decisions. While these mostly small correlations may seem surprising, they may be rooted in differences between intrinsic and extrinsic religious motivation. While it is intrinsically motivated religiosity that is assumed to exhibit functional equivalence with intelligence to a certain degree, the functions that religiosity fulfills may qualify it as extrinsically motivated (Zuckerman et al., 2013). This paradox could be resolved by distinguishing two forms of extrinsic religiosity: social extrinsic orientation (motivated by the attainment of social benefits) and personal extrinsic orientation (motivated by overcoming personal problems; Gorsuch and McPherson, 1989). While this distinction has been empirically demonstrated for Protestants, in other religious groups including Catholics, intrinsic religious orientations and personal extrinsic orientations appear to represent a single dimension that can be clearly distinguished from social extrinsic orientation (Flere and Lavric, 2008).

Although individual faith (in contrast to institutional, social, or ritual forms of religiosity) might be more pronounced in Protestants than in other denominations (Flere and Lavric, 2008), it can be assumed to be an important driver of religious behaviors as well. Religious rituals and practices being more important in Catholicism than in Protestantism could mean that Protestants engaging in religious behaviors do so out of deep personal conviction (e.g., Baptists attending a believer’s baptism). In other words: religious behaviors in Protestants might be an even better indicator for their personal beliefs than they are for Catholics. This would explain our observed more pronounced associations with intelligence for religious beliefs than for religious behaviors in Catholics but not in Protestants.

These results show that future research need to take denominations into account when examining religiosity associations with further variables. Moreover, potential interactions of religion with different societal values of being religious in different regions the world (e.g., Ritchie et al., 2014) may further moderate such relationships.

### Limitations and future directions

Some limitations of this study need to be acknowledged. First, the individual-level US data were collected in the 1970s and 1990s. Since then, institutionalized religiosity has been on a decline in the United States (Pew Research Center, 2019), thus possibly affecting associations with intelligence. However, results of our meta-analytic examinations broadly supported the findings of our primary data analyses. Second, Muslim (and in most cases Jewish) participants were underrepresented in our sample and could therefore not be accounted for in denomination-specific analyses. We therefore only assessed Catholics and Protestants from the United States in our primary data analysis approach. In addition, the studies included in our meta-analysis were predominantly conducted in Western, Christian countries (most of them in the US), thus making it impossible to examine effects of different religious denominations in our meta-analysis due to insufficient reporting of this variable.

Specifically, if the concept of God was primarily responsible for making intelligent people skeptical about religions, intelligence and religiosity associations can be expected to show different strengths in polytheistic religions (like Hinduism) or religions without the concept of God (like Buddhism; see Zuckerman et al., 2020). Therefore, future researchers may wish to examine the intelligence and religiosity link in such denominations. Similarly, there is virtually no evidence for this association for non-Christian denominations like Islam or Judaism, thus warranting targeted investigations.

Another limitation pertains to the suboptimal assessment standard for religiosity in the NLSY studies. Whilst cognitive abilities were in both studies assessed with extensively validated test instruments, religiosity was assessed with only few items (one in the NLSY79-cohort and five in the NLSY97-cohort). It can be argued that this approach provides a rather crude assessment of a comprehensive and multifaceted construct such as religious faith or a religious worldview. While in general single item measures may exhibit either unknown or inferior reliability or validity compared to scale scores (Allen et al., 2022), they have been shown to represent good indicators of religious attitudes (Abdel-Khalek, 2007). Moreover, we note that the use of single-item measures (e.g., Daws and Hampshire, 2017; Drewelies et al., 2018; Furnham and Grover, 2020; Hartman et al., 2017; Pollet and Schnell, 2017; Razmyar and Reeve, 2013; Shenhav et al., 2012; Sherkat, 2010, 2011; Ståhl and van Prooijen, 2018) or measures with few items (e.g., Erlandsson et al., 2018; Heaven et al., 2011; Hergovich and Arendasy, 2005; Nilsson et al., 2019; Pennycook et al., 2014) is common practice in the scientific literature. However, the validity and reliability of items assessing religiosity are supported by the strength of the intelligence and religiosity associations that we found here which are consistent with findings in the available literature (e.g., Lewis et al., 2011; Łowicki et al., 2020).

Nonetheless, we encourage the use of more refined religiosity assessments in further work. Future researchers may wish to use standardized psychometric scales to assess religiosity instead of mere single or dual item measures. Moreover, examining the relationship of intelligence and religiosity across societies with different social status of religiosity in respective societies (i.e., secular vs. orthodox) is necessary.

## Conclusion

Here, we provide evidence for stronger intelligence and religiosity associations for cognitive ability tasks with higher *g*-loadings based on primary- and meta-analytical data, thus tentatively supporting a causal pathway of intelligence influences on religiosity. Moreover, more pronounced associations of intelligence with religious beliefs than with religious behaviors in Catholics but not in Protestants suggest that the investigated associations may be differentiated according to religious denomination.

## Data Availability

The original contributions presented in the study are included in the article/Supplementary material, further inquiries can be directed to the corresponding author.

## Footnotes

1
https://www.oatd.org
