# Supplementary material for: The negative intelligence-religiosity link may be differentiated according to cognitive test g-loadings and (Christian) religious denominations: primary study and meta-analytical evidence
Source: Front Psychol. 2026 Mar 12;17:1633400. doi: 10.3389/fpsyg.2026.1633400 (PMC13017962; doi:10.3389/fpsyg.2026.1633400)
Supplement: Supplementary file 1 [file Data_Sheet_1.pdf]

- In the preregistration we stated that we will obtain an estimate of g for each participant: "The ability estimates of the twelve subtests of the CAT-ASVAB are going to be subjected to an unrotated principal component analysis. Then, relative frequencies of correct answers were obtained for each variable by computing the percentage of correct vs. incorrect answers respectively. These 12 scores were then multiplied with their respective factor loadings on the first factor resulting from the PCA and afterwards averaged (added to a total score, which was then divided by 10 or 12 respectively). Finally, this score was *z*-standardized by subtracting the sample mean and dividing the resulting value by the respective standard deviation. The result is considered as a proxy of *g*."
  As we do not need individual scores regarding *g*, but rather the g-loadings of the ASVAB or CAT-ASVAB subtests, we conducted only the PCA and used the loadings on the first unrotated factor as indicator for the respective subtest's loading on *g*.
- We stated in the preregistration that we will investigate time trends of religious beliefs & academic achievement associations (H8) as well of religious behavior & academic achievement associations (H9). However, the NLSY data set only provides academic achievement measures for 1981, making longitudinal data analyses impossible. The design of the study is therefore a between-subjects design (as opposed to a between- and within-subjects design).
- For hypotheses 5 and 6 we stated that "Formal tests for differences in strength will be conducted". Because of the large number of correlation coefficients, we decided to regress associations of the subtest scores and religiosity indices on *g*-loadings instead.
