# Supplementary material for: The negative intelligence-religiosity link may be differentiated according to cognitive test g-loadings and (Christian) religious denominations: primary study and meta-analytical evidence
Source: Front Psychol. 2026 Mar 12;17:1633400. doi: 10.3389/fpsyg.2026.1633400 (PMC13017962; doi:10.3389/fpsyg.2026.1633400)
Supplement: Supplementary file 2 [file Data_Sheet_2.pdf]

Supplement S2. Dichotomizing frequency of religious attendance (No attendance: *N* = 2543, attendance: *N* = 10126)

Table 1. Correlations of ASVAB subtests and religious attendance for the 1979 cohort.

	General Science	Arithmetic Reasoning	Word Knowledge	Paragraph Comprehension	Numerical Operations	Coding Speed	Auto and Shop Information	Mathematics Knowledge	Mechanical Comprehension	Electronics Info	Religious Attendance	Age
General Science												
Arithmetic Reasoning	.741***											
Word Knowledge	.818***	.737***										
Paragraph Comprehension	.711***	.697***	.797***									
Numerical Operations	.550***	.636***	.624***	.617***								
Coding Speed	.498***	.549***	.589***	.588***	.720***							
Auto and Shop Information	.653***	.552***	.569***	.480***	.358***	.313***						
Mathematics Knowledge	.716***	.819***	.714***	.687***	.644***	.553***	.450***					
Mechanical Comprehension	.726***	.698***	.650***	.585***	.451***	.408***	.734***	.623***				
Electronics Info	.781***	.678***	.721***	.628***	.468***	.417***	.744***	.614	.752***			
Religious Attendance	-.008	.030**	.009	.034***	.070***	.055***	-.089***	.058***	-.033***			
Age	.193***	.167***	.255***	.186***	.133***	.185***	.232***	.094***	.170***	.258***	-.088***	
Sex	-.148***	-.104***	.022*	.087***	.112***	.207***	-.447***	-.025**	-.308***	-.302***	.085***	.008

Note. Cell entries are Pearson correlation coefficients for the total sample of the NLSY79 cohort. Sex: 0 = men; 1 = women. Religious attendance = Frequency of religious attendance (0 = no attendance; 1 = attendance). *: $p < .05$, **: $p < .01$, ***: $p < .001$.

Table 2. Correlations of academic achievement scores and religious attendance for the 1979 cohort.

	SAT math	SAT verbal	ACT math	ACT verbal	PSAT math	PSAT verbal	Religious Attendance	Age
SATmath								
SATverbal	.748***							
ACTmath	.860***	.646***						
ACTverbal	.723***	.738***	.676***					
PSATmath	.863***	.701***	.823***	.648***				
PSATverbal	.660***	.860***	.616***	.724***	.713***			
Religious Attendance	-.114***	-.135***	-.087**	-.030	-.068*	-.061*		
Age	-.022	.030	.051	.037	.004	.048	-.088***	
Sex	-.260***	-.114***	-.222***	.025	-.196***	-.082**	.085***	.008

Note. Cell entries are Pearson correlation coefficients for the total sample of the NLSY79 cohort. Sex: 0 = men; 1 = women. SAT: Scholastic Aptitude Test, ACT: American College Test, PSAT: Preliminary Scholastic Aptitude Test. Religious attendance = Frequency of religious attendance (0 = no attendance; 1 = attendance). *: $p < .05$, **: $p <.01$, ***: $p < .001$.

Table 3. Correlations of intelligence measures and religious attendance for the 1979 cohort.

	California	Otis	Lorge	Henmon	Kuhlmann	DAT	Coop	Stanford	Wechsler	Religious Attendance	Age
California											
Otis	.309**										
Lorge	.592***	.605***									
Henmon	.205	.846***	.354								
Kuhlmann	.169	.729***	.849***	.270							
DAT	.666***	.369***	.340***	.268	.448						
Coop	-.096	.706***	.273	.666**	.224	.393*					
Stanford	.028	.389	-.101	.534*	.157	.861	.				
Wechsler	.698*	.780***	.075	.	.098	.839*	.	.806***			
Religious Attendance	.049	.026	-.002	-.043	.060	.052	.003	.055	-.141		
Age	.056	.062*	.022	.172*	.153*	-.047	.191*	-.117	.037	-.088***	
Sex	.005	.054	.133***	-.005	-.035	-.020	-.010	-.095	-.019	0.085***	.008

Note. Cell entries are Pearson correlation coefficients for the total sample of the NLSY79 cohort. Sex: 0 = men; 1 = women. Religious attendance = Frequency of religious attendance (0 = no attendance; 1 = attendance). California: California Test of Mental Maturity, Otis: Otis-Lennon Test of Mental Maturity, Lorge: Lorge-Thorndike Intelligence Test, Henmon: Henmon-Nelson Test of Mental Maturity, Kuhlmann: Kuhlmann-Anderson Intelligence Test, DAT: Differential Aptitude Test, Coop: Coop School & College Ability Test, Stanford: Stanford-Binet Intelligence Scale, Wechsler: Wechsler Intelligence Scale for Children. *: $p < .05$, **: $p <.01$, ***: $p < .001$.

Table 4. *g*-loadings of ASVAB and CAT_ASVAB subtests.

Subtest (ASVAB) 1979	*g*-loading	*r* frequency of rel. attendance	Subtest (CAT_ASVAB) 1997	*g*-loading	*r* Value	*r* Obey	*r* Decision	*r* with Happens	*r* Praying
General science	0.334	-.008	Mathematics knowledge	0.351	-.041	-.143	-.050	.050	.001
Word knowledge	0.333	.818***	General science	0.346	-.023	-.124	-.031	.060	.014
Paragraph comprehension	0.326	.711***	Arithmetic reasoning	0.346	-.013	-.132	-.006	.059	.032
Arithmetic reasoning	0.325	.741***	Paragraph comprehension	0.339	.031	-.032	-.023	.055	.036
Electronics info	0.322	.781***	Word knowledge	0.321	-.014	-.034	-.005	.027	.052
Mechanical comprehension	0.316	.726***	Assembling objects	0.296	.014	-.040	.003	.038	-.025
Mathematics knowledge	0.313	.716***	Mechanical comprehension	0.295	-.013	-.083	-.013	.022	.038
Numerical operations	0.303	.550***	Coding speed	0.273	.006	-.171	-.058	.082	-.055
Auto and shop information	0.301	.653***	Numerical operations	0.266	<.001	-.188	-.058	.092	-.048
Coding speed	0.286	.498***	Electronics info	0.250	-.056	-.077	-.075	.027	.002
			Shop information	0.165	-.059	-.117	-.039	.008	-.095
			Auto information	0.108	-.097	-.133	.042	-.091	.012

Note. The ASVAB-subtests are ordered descending in regards to the respective loading on the first factor of the principal component analysis. For the CAT-ASVAB, factor loadings were obtained via positive ability estimates. Value = "I don't need religion to have good values" (0 = True; 1 = False), Obey = "Religious teachings should be obeyed exactly as written in every situation" (0 = False; 1 = True), Decision = "I often ask God to help me make decisions" (0 = False; 1 = True), Happens = "God has nothing to do with what happens to me personally" (0 = True; 1 = False), Praying = "I pray more than once a day" (0 = False; 1 = True). frequency of rel. attendance: 0 = no attendance; 1 = attendance.
