# Supplementary material for: The negative intelligence-religiosity link may be differentiated according to cognitive test g-loadings and (Christian) religious denominations: primary study and meta-analytical evidence
Source: Front Psychol. 2026 Mar 12;17:1633400. doi: 10.3389/fpsyg.2026.1633400 (PMC13017962; doi:10.3389/fpsyg.2026.1633400)
Supplement: Supplementary file 3 [file Data_Sheet_3.pdf]

Supplement S4. Denomination-specific associations of intelligence and religiosity

Table 1. 1979

	General Science	Arithmetic Reasoning	Word Knowledge	Paragraph Comprehension	Numerical Operations	Coding Speed	Auto and Shop Information	Mathematics Knowledge	Mechanical Comprehension	Electronics Info	Average *r*
Catholics (*N* = 3899)											
Religious Attendance	-.009 (3593)	.031 (3587)	-.027 (3584)	.007 (3601)	.029 (3601)	.029 (3601)	-.116*** (3601)	.092*** (3601)	-.042* (3582)	-.091*** (3601)	-.010 (3595)
Protestants (*N* = 6674)											
Religious Attendance	-.066*** (6290)	-.033* (6283)	-.050*** (6287)	-.014 (6308)	-.003 (6308)	-.007 (6308)	-.154*** (6308)	.033* (6308)	-.078*** (6278)	-.116*** (6308)	-.049 (6299)
Jews (*N* = 110)											
Religious Attendance	.021 (102)	.127 (103)	.009 (102)	.033 (103)	.221* (103)	.082 (103)	.006 (103)	.051 (103)	.117 (103)	.027 (103)	.069 (103)
Muslims (*N* = 25)											
Religious Attendance	-.082 (21)	-.245 (21)	-.312 (21)	-.075 (21)	-.264 (21)	.103 (21)	-.327 (21)	-.441 (21)	-.265 (21)	-.399 (21)	-.227 (21)

Note. Cell entries are Pearson correlation coefficients of religious attendance and the respective ASVAB-subtest scores for the total sample of the NLSY79 cohort. Average *r* = mean Pearson correlation for religious attendance and all 10 ASVAB-subtests, respectively. *p<.05, **p<.01, ***p<.001

Table 2. Catholics 1997

	r Value (*N*)	*r* Obey (*N*)	*r* Decision (*N*)	*r* Happens (*N*)	*r* Praying (*N*)
General science	-.067 (432)	-.112* (431)	-.048 (432)	.052 (430)	.027 (431)
Arithmetic reasoning	-.015 (458)	-.137** (456)	.014 (458)	.121** (454)	.116* (456)
Word knowledge	-.020 (370)	-.037 (370)	.041 (370)	.057 (368)	.092 (366)
Paragraph comprehension	.016* (517)	-.029 (514)	-.058 (516)	.120** (512)	.094* (513)
Numerical operations	.036 (1389)	-.185*** (1382)	-.082** (1387)	.117*** (1383)	-.040 (1386)
Coding speed	.070* (1357)	-.175*** (1758)	-.037 (1355)	.129*** (1351)	.004 (1354)
Auto information	-.255 (27)	-.138 (1758)	-.118 (27)	.065 (27)	.081 (27)
Shop information	.017 (95)	-.191 (1758)	.096 (95)	.156 (95)	-.155 (95)
Mathematics knowledge	-.007 (618)	-.125** (1758)	-.014 (617)	.128** (613)	.081* (614)
Mechanical comprehension	-.092 (292)	-.089 (1350)	-.025 (292)	.125* (291)	.088 (291)
Electronics info	-.190* (143)	-.088 (143)	-.051 (143)	.138 (142)	.022 (142)
Assembling objects	.079 (468)	-.039 (466)	.054 (466)	.052 (464)	.073 (466)

Note. *p<.05, **p<.01, ***p<.001

Table 3. Protestants 1997

	r Value (*N*)	*r* Obey (*N*)	*r* Decision (*N*)	*r* Happens (*N*)	*r* Praying (*N*)
General science	-.010 (758)	-.335 (31)	-.023 (758)	.059 (752)	.041 (756)
Arithmetic reasoning	.004 (769)	-.065 (28)	.020 (768)	.040 (763)	.040 (765)
Word knowledge	.022 (613)	-.097 (27)	.035 (614)	.018 (610)	.087* (611)
Paragraph comprehension	.065 (863)	-.157 (35)	.045 (862)	.060 (856)	.068* (858)
Numerical operations	-.005 (2541)	.048 (99)	-.031 (2540)	.101*** (2530)	-.042* (2533)
Coding speed	<.001 (2441)	.134 (98)	-.012 (2440)	.078*** (2430)	-.056** (2433)
Auto information	-.136 (77)	.823 (3)	.099 (77)	-.115 (76)	-.035 (76)
Shop information	-.080 (171)	.437 (7)	-.055 (171)	-.131 (171)	-.051 (171)
Mathematics knowledge	-.068* (985)	-.247 (33)	-.039 (984)	.034 (979)	-.010 (979)
Mechanical comprehension	.049 (490)	-.065 (22)	.025 (490)	.009 (486)	.037 (488)
Electronics info	-.039 (270)	.059 (12)	-.064 (270)	-.002 (267)	-.004 (267)
Assembling objects	-.021 (744)	.047 (29)	-.009 (744)	.050 (739)	-.062 (740)

Note. *p<.05, **p<.01, ***p<.001

Table 4. Jews 1997

	r Value (*N*)	*r* Obey (*N*)	*r* Decision (*N*)	*r* Happens (*N*)	*r* Praying (*N*)
General science	.315 (27)	NA	.011 (27)	.131 (27)	NA
Arithmetic reasoning	.233 (27)	NA	.050 (27)	-.023 (27)	-.204 (27)
Word knowledge	.342 (29)	NA	-.250 (29)	.172 (29)	-.168 (29)
Paragraph comprehension	.422* (31)	NA	.122 (31)	.197 (31)	-.059 (31)
Numerical operations	.115 (45)	NA	.157 (45)	.074 (44)	-.100 (45)
Coding speed	.124 (44)	NA	-.058 (44)	.024 (43)	-.205 (44)
Auto information	-.487 (3)	NA	NA	NA	NA
Shop information	.809 (6)	NA	NA	.399 (6)	NA
Mathematics knowledge	.196 (33)	NA	.057 (33)	.145 (33)	-.301 (33)
Mechanical comprehension	.117 (12)	NA	-.035 (12)	.017 (2)	NA
Electronics info	.190 (8)	NA	-.326 (8)	.384 (8)	NA
Assembling objects	.476 (17)	NA	-.161 (17)	.310 (17)	.064 (17)

Note. *p<.05, **p<.01, ***p<.001

Table 5. Muslims 1997

	r Value (*N*)	*r* Obey (*N*)	*r* Decision (*N*)	*r* Happens (*N*)	*r* Praying (*N*)
General science	-.628 (6)	NA	NA	.229 (6)	-.045 (6)
Arithmetic reasoning	-.315 (12)	NA	.317 (12)	-.025 (12)	.216 (12)
Word knowledge	-398 (4)	NA	NA	.308 (4)	.308 (4)
Paragraph comprehension	-.622 (8)	NA	NA	-.567 (8)	-.567 (8)
Numerical operations	-.069 (27)	NA	.077 (27)	.216 (27)	-.081 (27)
Coding speed	-.304 (26)	NA	.091 (26)	.235 (26)	-.142 (26)
Auto information	NA	NA	NA	NA	NA
Shop information	NA	NA	NA	NA	NA
Mathematics knowledge	-.262 (11)	NA	NA	.214 (11)	.214 (11)
Mechanical comprehension	-.308 (4)	NA	NA	NA	NA
Electronics info	-.947 (3)	NA	NA	-.752 (3)	-.752 (3)
Assembling objects	.082 (7)	NA	NA	-.273 (7)	-.273 (7)

Note. *p<.05, **p<.01, ***p<.001
