# Supplementary material for: The negative intelligence-religiosity link may be differentiated according to cognitive test g-loadings and (Christian) religious denominations: primary study and meta-analytical evidence
Source: Front Psychol. 2026 Mar 12;17:1633400. doi: 10.3389/fpsyg.2026.1633400 (PMC13017962; doi:10.3389/fpsyg.2026.1633400)
Supplement: Supplementary file 4 [file Data_Sheet_4.pdf]

Table 1. Formal tests for differences in strength of religious attendance associations with all ASVAB-subtests.

	Correlation with frequency of rel. attendance (*N*)	z_1	z_2	z_3	z_4	z_5	z_6	z_7	z_8	z_9
General Science	-.052*** (11833)	-								
Arithmetic Reasoning	-.014 (11819)	2.891**	-							
Word Knowledge	-.042*** (11819)	0.760	-2.130*	-						
Paragraph Comprehension	-.006 (11863)	3.522***	0.628	2.758**	-					
Numerical Operations	.023* (11863)	5.761***	2.866**	4.998***	2.240*	-				
Coding Speed	.024* (11863)	5.807***	2.913**	5.045***	2.287*	0.047	-			
Auto and Shop Information	-.147*** (11863)	-7.407***	-10.297***	-8.165***	-10.935***	-13.176***	-13.222***	-		
Mathematics Knowledge	.044*** (11863)	7.370***	4.476***	6.608***	3.851***	1.611	1.564	14.786***	-	
Mechanical Comprehension	-.071*** (11810)	-1.458	-4.347***	-2.218*	-4.979***	-7.217***	-7.263***	5.944***	-8.826***	-
Electronics Info	-.110*** (11863)	-4.508***	-7.399***	-5.267***	-8.034***	-10.275***	-10.321***	2.901**	-11.885***	-3.046**

Note. In the leftmost column, Pearson correlations between frequency of religious attendance and scores on the respective ASVAB subtest for the total sample of the NLSY79 cohort are provided. Pearson coefficients allow for formal comparisons of correlation coefficient strengths. *z*-columns represent test statistics for such comparisons between correlations of religiosity with different ASVAB-subtests. Numbers in parentheses represent the respective sample sizes. *: $p < .05$; **: $p < .01$; ***: $p < .001$.

Table 2. Formal tests for differences in strength of “I don't need religion to have good values” associations with all CAT-ASVAB-subtests.

	Correlation with relval(N)	z_1	z_2	z_3	z_4	z_5	z_6	z_7	z_8	z_9	z_{10}	z_{11}
General Science	-.023 (1491)	-										
Arithmetic Reasoning	-.013 (1551)	0.283	-									
Word Knowledge	-.014 (1271)	0.243	-0.025	-								
Paragraph Comprehension	.031 (1755)	1.520	1.242	1.201	-							
Numerical Operations	<.001 (4802)	0.773	0.434	0.432	-1.098	-						
Coding Speed	.006 (4646)	0.957	0.622	0.606	-0.894	0.271	-					
Auto Information	-.097 (136)	-0.819	-0.934	-0.915	-1.420	-1.104	-1.167	-				
Shop Information	-.059 (336)	-0.593	-0.765	-0.735	-1.498	-1.039	-1.136	0.372	-			
Mathematics Knowledge	-.041 (2006)	-0.515	-0.824	-0.750	-2.177**	-1.525	-1.726	0.631	0.310	-		
Mechanical Comprehension	-.013 (1007)	0.238	-0.014	0.010	-1.109	-0.381	-0.540	0.909	0.722	0.707	-	
Electronics Info	-.056 (515)	-0.649	-0.854	-0.813	-1.729	-1.209	-1.326	0.420	0.038	-0.316	-0.791	-
Assembling Objects	.014 (1522)	0.771	0.495	0.495	-0.726	0.176	-0.013	1.131	1.059	1.344	0.453	1.201

Note. In the leftmost column, Pearson correlations between “I don't need religion to have good values” (0 = True; 1 = False) and scores on the respective ASVAB subtest for the total sample of the NLSY97 cohort are provided. Pearson coefficients allow for formal comparisons of correlation coefficient strengths. *z*-columns represent test statistics for such comparisons between correlations of religiosity with different ASVAB-subtests. Numbers in parentheses represent the respective sample sizes. **: $p < .01$.

Table 3. Formal tests for differences in strength of "Religious teachings should be obeyed exactly as written in every situation" associations with all CAT-ASVAB-subtests.

	Correlation with obey (N)	z_1	z_2	z_3	z_4	z_5	z_6	z_7	z_8	z_9	z_{10}	z_{11}
General Science	-.124 (471)	-										
Arithmetic Reasoning	-.132 (500)	-0.122	-									
Word Knowledge	-.034 (405)	1.335	1.471	-								
Paragraph Comprehension	-.032 (564)	1.486	1.638	0.034	-							
Numerical Operations	-.188 (1540)	-1.233	-1.109	-2.783**	-3.205**	-						
Coding Speed	-.171 (1505)	-0.891	-0.760	-2.458*	-2.834**	0.493	-					
Auto Information	-.133 (31)	-0.047	-0.006	-0.511	-0.527	0.294	0.200	-				
Shop Information	-.117 (102)	0.068	0.139	-0.743	-0.785	0.700	0.527	0.077	-			
Mathematics Knowledge	-.143 (664)	-0.313	-0.186	-1.735	-1.950	0.992	0.606	-0.051	-0.245	-		
Mechanical Comprehension	-.083 (324)	0.581	0.697	-0.651	-0.728	1.746	1.452	0.260	0.301	0.897	-	
Electronics Info	-.077 (157)	0.511	0.600	-0.457	-0.501	1.332	1.119	0.275	0.311	0.742	0.055	-
Assembling Objects	-.040 (509)	1.332	1.477	-0.081	-0.125	2.936**	2.580**	0.487	0.709	1.766	0.607	0.603

Note. In the leftmost column, Pearson correlations between "Religious teachings should be obeyed exactly as written in every situation" (0 = False; 1 = True) and scores on the respective ASVAB subtest for the total sample of the NLSY97 cohort are provided. Pearson coefficients allow for formal comparisons of correlation coefficient strengths. *z*-columns represent test statistics for such comparisons between correlations of religiosity with different ASVAB-subtests. Numbers in parentheses represent the respective sample sizes. *: $p < .05$; **: $p < .01$; ***.

Table 4. Formal tests for differences in strength of "I often ask God to help me make decisions" associations with all CAT-ASVAB-subtests.

	Correlation with dec(N)	z_1	z_2	z_3	z_4	z_5	z_6	z_7	z_8	z_9	z_{10}	z_{11}
General Science	-.031 (1491)	-										
Arithmetic Reasoning	-.006 (1550)	0.684	-									
Word Knowledge	-.005 (1272)	0.683	0.033	-								
Paragraph Comprehension	-.023 (1752)	0.228	-0.482	-0.490	-							
Numerical Operations	-.058 (4799)	-0.922	-1.785	-1.693	-1.267	-						
Coding Speed	-.058 (4643)	-0.935	-1.795	-1.703	-1.279	-0.025	-					
Auto Information	.042 (136)	0.802	0.529	0.510	0.718	1.137	1.142	-				
Shop Information	-.039 (336)	-0.135	-0.546	-0.556	-0.271	0.339	0.347	-0.787	-			
Mathematics Knowledge	-.050 (2003)	-0.571	-1.311	-1.272	-0.843	0.293	0.311	-1.029	-0.193	-		
Mechanical Comprehension	-.013 (1006)	0.438	-0.171	-0.194	0.249	1.304	1.314	-0.593	0.412	0.968	-	
Electronics Info	-.075 (515)	-0.872	-1.363	-1.351	-1.049	-0.372	-0.361	-1.205	-0.519	-0.507	-1.152	-
Assembling Objects	.003 (1520)	0.914	0.235	0.191	0.721	2.060*	2.069*	-0.434	0.686	1.553	0.379	1.526

Note. In the leftmost column, Pearson correlations between "I often ask God to help me make decisions" (0 = False; 1 = True) and scores on the respective ASVAB subtest for the total sample of the NLSY97 cohort are provided. Pearson coefficients allow for formal comparisons of correlation coefficient strengths. z-columns represent test statistics for such comparisons between correlations of religiosity with different ASVAB-subtests. Numbers in parentheses represent the respective sample sizes. *: $p < .05$.

Table 5. Formal tests for differences in strength of "God has nothing to do with what happens to me personally" associations with all CAT-ASVAB-subtests.

	Correlation with hap(N)	z_1	z_2	z_3	z_4	z_5	z_6	z_7	z_8	z_9	z_{10}	z_{11}
General Science	.060 (1483)	-										
Arithmetic Reasoning	.059 (1540)	-0.019	-									
Word Knowledge	.027 (1266)	-0.852	-0.842	-								
Paragraph Comprehension	.055 (1742)	-0.135	-0.117	0.754	-							
Numerical Operations	.092 (4782)	1.098	1.137	2.064*	1.336	-						
Coding Speed	.082 (4626)	0.758	0.792	1.741	0.974	-0.486	-					
Auto Information	-.091 (135)	-1.666	-1.660	-1.297	-1.623	-2.085*	-1.970	-				
Shop Information	.008 (336)	-0.856	-0.847	-0.313	-0.788	-1.492	-1.313	0.966	-			
Mathematics Knowledge	.050 (1993)	-0.268	-0.250	0.652	-0.135	-1.568	-1.187	1.581	0.721	-		
Mechanical Comprehension	.022 (1000)	-0.927	-0.918	-0.126	-0.836	-2.029*	-1.736	1.223	0.220	-0.742	-	
Electronics Info	.027 (511)	-0.643	-0.633	-0.008	-0.561	-1.408	-1.191	1.210	0.267	-0.480	0.091	-
Assembling Objects	.038 (1512)	-0.596	-0.583	0.285	-0.484	-1.844	-1.498	1.427	0.497	-0.369	0.397	0.220

Note. In the leftmost column, Pearson correlations between "God has nothing to do with what happens to me personally" (0 = True; 1 = False) and scores on the respective ASVAB subtest for the total sample of the NLSY97 cohort are provided. Pearson coefficients allow for formal comparisons of correlation coefficient strengths. *z*-columns represent test statistics for such comparisons between correlations of religiosity with different ASVAB-subtests. Numbers in parentheses represent the respective sample sizes. *: $p < .05$.

Table 6. Formal tests for differences in strength of "I pray more than once a day" associations with all CAT-ASVAB-subtests.

	Correlation with pray(N)	z_1	z_2	z_3	z_4	z_5	z_6	z_7	z_8	z_9	z_{10}	z_{11}
General Science	.014 (1488)	-										

Arithmetic Reasoning	.032 (1545)	0.497	-									
Word Knowledge	.052 (1265)	0.981	0.513	-								
Paragraph Comprehension	.036 (1745)	0.632	0.121	-0.412	-							
Numerical Operations	-.048 (4791)	-2.075*	-2.722**	-3.134**	-3.000**	-						
Coding Speed	-.055 (4635)	-2.301*	-2.949**	-3.343***	-3.236**	-0.339	-					
Auto Information	.012 (135)	-0.021	-0.220	-0.431	-0.268	0.677	0.756	-				
Shop Information	-.095 (336)	-1.799	-2.104*	-2.380*	-2.197*	-0.837	-0.713	-1.042	-			
Mathematics Knowledge	.001 (1995)	-0.393	-0.930	-1.418	-1.091	1.806	2.058*	-0.129	1.615	-		
Mechanical Comprehension	.038 (1003)	0.598	0.158	-0.309	0.054	2.477*	2.670**	0.285	2.111*	0.980	-	
Electronics Info	.002 (511)	-0.224	-0.579	-0.934	-0.671	1.074	1.222	-0.099	1.383	0.039	-0.661	-
Assembling Objects	-.025 (1516)	-1.077	-1.587	-2.017*	-1.755	0.755	0.989	-0.413	1.152	-0.759	-1.566	-0.543

Note. In the leftmost column, Pearson correlations between “I pray more than once a day” (0 = False; 1 = True) and scores on the respective ASVAB subtest for the total sample of the NLSY97 cohort are provided. Pearson coefficients allow for formal comparisons of correlation coefficient strengths. *z*-columns represent test statistics for such comparisons between correlations of religiosity with different ASVAB-subtests. Numbers in parentheses represent the respective sample sizes. *: $p < .05$; **: $p < .01$; ***: $p < .001$.
