# Supplementary material for: The negative intelligence-religiosity link may be differentiated according to cognitive test g-loadings and (Christian) religious denominations: primary study and meta-analytical evidence
Source: Front Psychol. 2026 Mar 12;17:1633400. doi: 10.3389/fpsyg.2026.1633400 (PMC13017962; doi:10.3389/fpsyg.2026.1633400)
Supplement: Supplementary file 5 [file Data_Sheet_5.pdf]

Supplement S5. Additional information on specification curve analysis and combinatorial meta-analysis

Multiverse and specification curve analyses

We specified the following "Which" factors: (i) type of religiosity assessment (beliefs, behaviors, mixed, either), (ii) sample type (pre-college, college, general population, either), (iii) status of publication (published, unpublished, either), (iv) *g*-loadedness (poor, fair, good, excellent, not specified, either). These specifications therefore yielded 4*4*3*6 = 288 possible combinations. We specified the following "How" factors: (i) effect metric (i.e., synthesis of Pearson *r*s vs. *z*-transformed coefficients) and (ii) estimator type (i.e., random-effects DerSimonian-Laird estimators vs. random-effects restricted maximum-likelihood estimators vs. fixed-effect estimators vs. unweighted estimation), yielding 3* 4 =12 different ways to analyze the data. In all, data and analysis specifications yielded 12*288 = 3,456 ways to include and analyze the data. Analyses were restricted to unique combinations that comprised at least two studies.

Inferential test of the specification-curve analysis

Study features for all primary studies were regarded as fixed, while new effect sizes were randomly assigned under the assumption of a true nill effect. This is achieved by drawing values from a normal distribution with an expected value of zero and a varying standard deviation corresponding to the respective sample's observed standard error (which was obtained via a fixed-effect model). This procedure was conducted 1,000 times in total. Based on all resulting bootstrapped specification curves the pointwise lower (2,5%) and upper (97,5%) limits for each specification number were obtained. These limits represent the confines of the null hypothesis: Limits not including zero are considered to be indicative of a non-nill summary effect.

Combinatorial meta-analysis

Because of the large number of possible unique subsets (2^{126}-1 = 8,507,059,173,023,462*10^{37}), we drew a random sample of 100,000 different data subsets and estimated their summary effects following well-established approaches (Voracek et al., 2019). To meaningfully assess outlier influences, studies with the smallest and largest effects were oversampled.
