# Supplementary material for: The negative intelligence-religiosity link may be differentiated according to cognitive test g-loadings and (Christian) religious denominations: primary study and meta-analytical evidence
Source: Front Psychol. 2026 Mar 12;17:1633400. doi: 10.3389/fpsyg.2026.1633400 (PMC13017962; doi:10.3389/fpsyg.2026.1633400)
Supplement: Supplementary file 6 [file Data_Sheet_6.pdf]

Supplement S6. Random-effects estimates without influential cases.

	Summary effect (*r*)	*SE*	95%*CI*	*Q*	I^2
Without 20 (*k* = 125)	-.130***	0.0122	[-0.154, -0.107]	1844.6084***	96.06%
Without 46 (*k* = 125)	-.131***	0.0126	[-0.156, -0.107]	1786.2969***	96.31%
Without 110 (*k* = 125)	-.138***	0.0125	[-0.162, -0.114]	1761.0209***	96.208%
Without 111 (*k* = 125)	-.139***	0.0123	[-0.162, -0.115]	1758.8651***	96.09%
Without 20,46 (*k* = 124)	-.127***	0.0118	[-0.150, -0.104]	1739.7382***	95.71%
Without 20,110 (*k* = 124)	-.134***	0.0117	[-0.156, -0.111]	1714.7779***	95.59%
Without 20,111 (*k* = 124)	-.134***	0.0115	[-0.157, -0.112]	1712.6069***	95.45%
Without 20,46,110 (*k* = 123)	-.131***	0.0113	[-0.152, -0.109]	1610.7661***	95.13%
Without 20,46,111 (*k* = 123)	-.131***	0.0111	[-0.152, -0.109]	1608.5111***	94.95%
Without 20,46,110,111 (*k* = 122)	-.135***	0.0103	[-0.155, -0.115]	1478.7953***	94.00%
Without 46,110 (*k* = 124)	-.135***	0.0121	[-0.158, -0.112]	1657.1701***	95.86%
Without 46,111 (k = 124)	-.135***	0.0119	[-0.158, -0.112]	1654.9303***	95.72%
Without 46,110,111 (*k* = 123)	-.139***	0.0112	[-0.160, -0.117]	1525.0593***	94.99%
Without 110,111 (*k* = 124)	-.142***	0.0117	[-0.165, -0.120]	1628.1346***	95.54%

Note. SD=standard deviation; 95% CI = 95% lower and upper bound of 95% confidence interval; Q = Cochran`s Q test statistic for heterogeneity; I^2 = ratio between true heterogeneity and total observed variation. ** p < .01, *** p < .001

20 = Southern & Plant (1968)

46 = McCullough, Enders, Brion, Jain (2005)

110 = (1) Ramírez?Luzuriaga, Ochaeta, Ramírez?Zea, DiGirolamo, et al. (2021)

111 = (2) Ramírez?Luzuriaga, Ochaeta, Ramírez?Zea, DiGirolamo, et al. (2021)
