# Supplementary material for: The negative intelligence-religiosity link may be differentiated according to cognitive test g-loadings and (Christian) religious denominations: primary study and meta-analytical evidence
Source: Front Psychol. 2026 Mar 12;17:1633400. doi: 10.3389/fpsyg.2026.1633400 (PMC13017962; doi:10.3389/fpsyg.2026.1633400)
Supplement: Supplementary file 7 [file Data_Sheet_7.pdf]

Author	Year	*N*	Effect sizes (*r*)	Intelligence measure
Dodrill	1976	44	.03 (verbal IQ), .09 (performance IQ)	WAIS
Clark	2004	77	-0.117 (verbal), -0.089 (performance)	WAIS III
Cottone, Drucker, Javier	2007	123	−0.10 (Comprehension), −0.03 (Similarities)	WAIS III comprehension and similarities and GPA
Heaven, Ciarrochi, Leeson	2011	375	−.08 (verbal ability), −.10 (numerical ability)	g of six numerical and three verbal tests
Shenav, Rand, Greene	2011	321	-.047 (matrix), -.081 (vocabulary)	Shipley Vocabulary Test, and WAIS III Matrix Reasoning Test
(1)Pennycook, Cheyne, Seli, Koehler, Fugelsang	2012	223	-.24 (WS & beliefs), -.15 (BRN & beliefs)	Assorted tests (WordSum, base-rate neutral)
(2)Pennycook, Cheyne, Seli, Koehler, Fugelsang	2012	267	-.13 (WS & beliefs), -.22 (BRN & beliefs)	Assorted tests (WordSum, base-rate neutral)
Sacher	2015	44	-0.23 (vocabulary), -0.28 (abstraction)	Shipley-2 abbreviated test of intelligence (vocabulary and abstract reasoning)
Pennycook, Ross, Koehler, Fugelsang	2016	1065	-.11 (numeracy), -.17 (wordsum)	Numeracy, WordSum
Hartman, Dieckmann, Sprenger, Stastny, DeMarree	2017	598	-.28 (fluid), -.35 (crystallized), -.14 (numeracy)	Numeracy, Shipley 2 tests
Perales	2018	11654	-.14 (NART), -.23 (SDMT), -.09 (BDS)	Verbal (spelling), matching symbols, and memory test
(2) Lowicki, Zajenkowski, van der Linden	2019	200	− .03 (Catell), − .01 (numbers), − .08 (paper folding)	Catell`s Culture Fair Intelligence Test 3, Number Series Test, Paper Folding Test
Šrol	2020	397	-.26 (numeracy), -.23 (matrix test)	Vienna matrix test (VMT)- short version + Berlin numeracy test & general and expanded numeracy questionnaire (Lipkus, Samsa, & Rimer, 2001)
Dutton & Kirkegaard	2021	4462	− 0.23, − 0.18, − 0.17, − 0.20, − 0.21, − 0.20, − 0.17, − 0.17, − 0.20, − 0.21, − 0.11, − 0.12, − 0.08, − 0.08, − 0.08, − 0.06, − 0.09, − 0.08, − 0.08	Grooved Pegboard Test, Paced Auditory Serial Addition Test, Rey-Osterrieth Complex Figure Drawing, Wechsler Adult Intelligence Scale-Revised (general knowledge & spatial ability), Word List Generation Test, Wisconsin Card Sort Test, Wide Range Achievement Test, California Verbal Learning Test, Army Classification Battery (verbal & arithmetic reasoning), Pattern Analysis Test, General Information Test, Armed Forces Qualification Test
Lace & Evans	2022	432	−0.19 (verbal reasoning), −0.11 (pattern recognition), −0.21 (matrix reasoning), −0.13 (Three-dimensional Rotation)	International Cognitive Ability Resource Sample Test (ICAR-16) - pattern recognition, nonverbal abstract reasoning, general knowledge and logic and visuospatial/perceptual ability
Borella, Ghisletta, Carbone, Aichele	2023	235	0.054 (vocabulary), − 0.182 (Raven`s)	Wechsler adult intelligence scale revised—vocabulary Subtest + Raven’s (fluid intelligence)
